# Xinfeng capsule improves hyperinflammation-associated hypercoagulability and self-perception in osteoarthritis by regulating KLF4 through METTL14-mediated m^6^A modification of lncRNA MEG3

**DOI:** 10.3389/fimmu.2026.1749727

**Published:** 2026-01-29

**Authors:** Mingyu He, Jian Liu, Yanqiu Sun, Yanyan Fang, Fanfan Wang

**Affiliations:** First Affiliated Hospital of Anhui University of Traditional Chinese Medicine, Hefei, China

**Keywords:** hyperinflammation- associated hypercoagulability, lncRNA MEG3, METTL14, osteoarthritis, Xinfeng capsule

## Abstract

**Background:**

Our previous studies have demonstrated that Xinfeng Capsule (XFC) exerts therapeutic effects on hyperinflammation-associated hypercoagulability and self-perception of patients (SPP) with osteoarthritis (OA). However, the underlying molecular mechanisms remain unclear.

**Objective:**

This study aimed to explore the mechanism by which XFC improves hyperinflammation-associated hypercoagulability and SPP in OA.

**Methods:**

A keyword co-occurrence network was constructed to identify key targets involved in hyperinflammation-associated hypercoagulability in OA patients. m6A prediction databases and RNA pull-down assays were used to identify potential m6A modification sites and key binding proteins of lncRNA MEG3. Peripheral blood mononuclear cells (PBMCs) were collected from OA patients and healthy controls, and global m^6^A levels were measured using a colorimetric assay. Methylated RNA immunoprecipitation quantitative polymerase chain reaction (MeRIP-qPCR) was used to detect m6A modification levels of lncRNA MEG3. Reverse transcription quantitative polymerase chain reaction (RT-qPCR) and Western blot (WB) were performed to determine the mRNA and protein expression levels of target genes. Enzyme-linked immunosorbent assay (ELISA) was used to measure inflammatory and coagulation-related factors. Finally, clinical data mining was conducted to clarify the clinical efficacy of XFC in improving hyperinflammation-associated hypercoagulability and SPP in OA patients, and *in vitro* experiments were performed to validate the underlying mechanisms.

**Results:**

The keyword co-occurrence network analysis indicated that inflammatory factors [interleukin-6 (IL-6), IL-10, and tumor necrosis factor-α (TNF-α)] and coagulation-related factors [tissue factor (TF), plasminogen activator inhibitor-1 (PAI-1), and prostaglandin I_2_ (PGI_2_)] are involved in the regulation of hyperinflammation-associated hypercoagulability in OA patients. PBMC analysis further confirmed these findings. In addition, the expression levels of lncRNA MEG3 and its target gene KLF4 were significantly decreased in OA patients and were closely associated with clinical indicators of inflammation, coagulation, and SPP. Methyltransferase-like 14 (METTL14) expression was significantly increased in OA patients and was negatively correlated with lncRNA MEG3 expression. Clinical data mining revealed that XFC is a key therapeutic agent for improving hyperinflammation-associated hypercoagulability and SPP in OA patients. XFC treatment reduced the expression levels of METTL14, pro-inflammatory factors, and procoagulant factors, while increasing the levels of lncRNA MEG3, KLF4, anti-inflammatory factors, and anticoagulant factors. *In vitro* These findings were further validated by *in vitro* experiments.

**Conclusion:**

This study indicates that XFC may upregulate the expression of lncRNA MEG3 and KLF4 by modulating METTL14-mediated m6A modification of lncRNA MEG3. Through this mechanism, XFC may regulate inflammatory responses and coagulation disorders, thereby improving SPP and exerting therapeutic effects on hyperinflammation-associated hypercoagulability in patients with OA.

## Graphical Abstract

**Figure:**
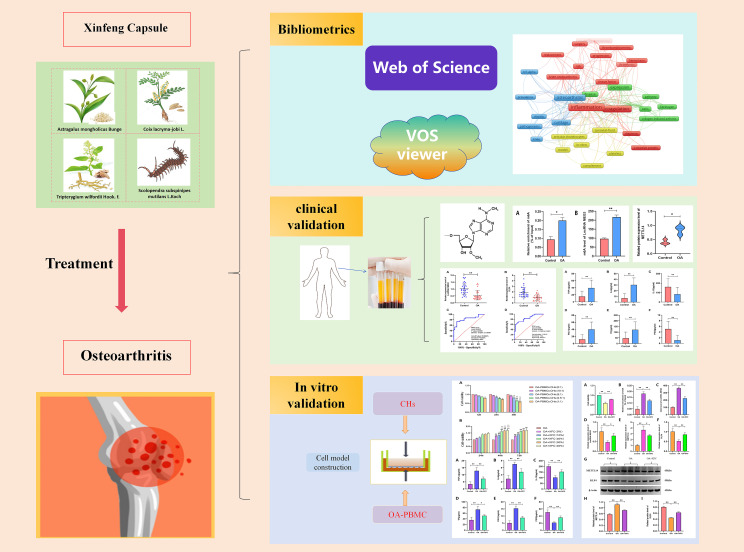

## 1 Introduction

Osteoarthritis (OA) is a leading cause of chronic disability in adults, primarily resulting from pain and joint dysfunction caused by characteristic pathological changes in joint tissues (1). This disease mainly manifests as joint pain, stiffness, and limited mobility (2). The prevalence of OA is approximately 50% in individuals over 60 years of age and increases to as high as 80% among those over 75 years old, with a disability rate of 53% (3). Although OA was once considered a simple “wear-and-tear “ disease, it is now recognized as being characterized at the molecular level by the presence of abundant pro-inflammatory mediators (4). These pro-inflammatory factors drive the production of proteolytic enzymes, leading to degradation of the extracellular matrix and subsequent damage to joint tissues (5). In OA, long-term chronic inflammation, cytokine imbalance, and immune dysfunction can induce vascular endothelial cell injury, thereby directly or indirectly activating the coagulation-fibrinolysis system and impairing anticoagulant mechanisms. This process ultimately results in microcirculatory disturbances, systemic circulatory abnormalities, and the development of a hypercoagulable state (6, 7). Therefore, suppressing inflammation and maintaining fibrinolytic balance has emerged as a promising strategy for improving hyperinflammation-associated hypercoagulability in patients with OA.

N^6^-methyladenosine (m^6^A) is the most abundant and reversible post-transcriptional modification in eukaryotic RNA and plays a critical role in regulating cellular pathophysiological functions by modulating RNA splicing, stability, and translation. It has been shown to be closely associated with a wide range of diseases, including OA (8–10). The dynamic regulation of m^6^A modification involves writers (methyltransferases), erasers (demethylases), and readers (m^6^A-binding proteins). Disruption or dysregulation of this dynamic process may impair cellular regulatory mechanisms and ultimately lead to the development of various diseases (11, 12). Therefore, an in-depth investigation of the roles, functions, and regulatory mechanisms of lncRNA m^6^A modification in OA may contribute to a more comprehensive understanding of OA pathogenesis and provide novel insights and therapeutic targets for its clinical diagnosis and treatment. Long noncoding RNA maternal expression gene 3 (lncRNA MEG3) is an imprinted lncRNA located at the DLK1-MEG3 locus on human chromosome 14q32.3 (13). Previous studies have demonstrated that lncRNA MEG3 plays a crucial role in the initiation and progression of OA by influencing chondrocyte (CH) proliferation and apoptosis, extracellular matrix (ECM) degradation, angiogenesis, and inflammatory responses in CHs (14, 15). Our previous research confirmed that downregulation of lncRNA MEG3 in OA promotes the expression of vascular endothelial growth factor (VEGF) through activation of the NF-κB signaling pathway, thereby exacerbating thrombosis and inflammatory responses (16). However, whether m^6^A modification contributes to the dysregulation of lncRNA MEG3 expression in patients with OA remains unclear.

Although extensive research has investigated various aspects of OA pathogenesis, progress in the development of novel therapeutic agents remains limited. Current treatment strategies still face substantial challenges in delaying disease progression and improving long-term prognosis. Consequently, the hospitalization rate, economic burden, and disability rate among patients with OA remain high (17). In recent years, traditional Chinese medicine (TCM) has gained increasing recognition as an important alternative pharmacotherapy for OA because of its relatively mild side effects and favorable therapeutic efficacy (18, 19). As a Chinese patent medicine, Xinfeng Capsule (XFC) has demonstrated notable clinical efficacy in alleviating the hypercoagulable state in OA patients by suppressing inflammatory responses (20).

XFC is an in-hospital preparation developed by the First Affiliated Hospital of Anhui University of Traditional Chinese Medicine (Anhui Medicine Production No. Z20050062; Patent No. ZL201310011369.8) and is composed of *Astragalus membranaceus*, Coix seed, centipede, and *Tripterygium wilfordii*. Previous studies have shown that XFC downregulates pro-inflammatory factors [interleukin (IL)-1], upregulates anti-inflammatory factors (IL-10), and improves hemorheological parameters as well as thromboxane/prostacyclin balance, thereby alleviating endothelial dysfunction in OA (21). To ensure effective quality control and quantitative analysis of the main active components of XFC, a characteristic chromatographic profile of the alcohol extract of XFC was established using high-performance liquid chromatography (HPLC). Three major active constituents, including anthocyanins, flavonoids, and flavonoid glycosides, were identified based on chemical composition spectra. The HPLC characteristic fingerprints of 10 batches of XFC exhibited a similarity greater than 0.90, indicating stable quality, a reliable manufacturing process, and minimal batch-to-batch variation (22–24). In addition, the clinical efficacy and safety of XFC in OA treatment were evaluated in a multicenter, double-blind, randomized controlled clinical trial. The results demonstrated that XFC was superior to glucosamine (GS) in improving total Western Ontario and McMaster Universities Arthritis Index (WOMAC) scores, WOMAC subscale scores for pain, stiffness, and physical function, visual analogue scale scores, Lequesne index scores, and 36-Item Short Form Survey scores, with no significant adverse reactions observed (25). These findings underscore the effectiveness and safety of XFC in the treatment of OA.

Previous studies have demonstrated the strong therapeutic effects of XFC in patients with OA. Nevertheless, the effects and underlying mechanisms by which XFC ameliorates the hypercoagulable state in OA through suppression of inflammation and endothelial dysfunction remain unclear. Therefore, this study integrates analyses of public databases, clinical peripheral blood assessments from patients, and *in vitro* experimental validation to further investigate the potential mechanisms by which lncRNA MEG3 regulates inflammation and coagulopathy in OA from the perspective of lncRNA m^6^A modification, as well as the interventional effects of XFC (**Graphical Abstract**).

**Figure 1:**
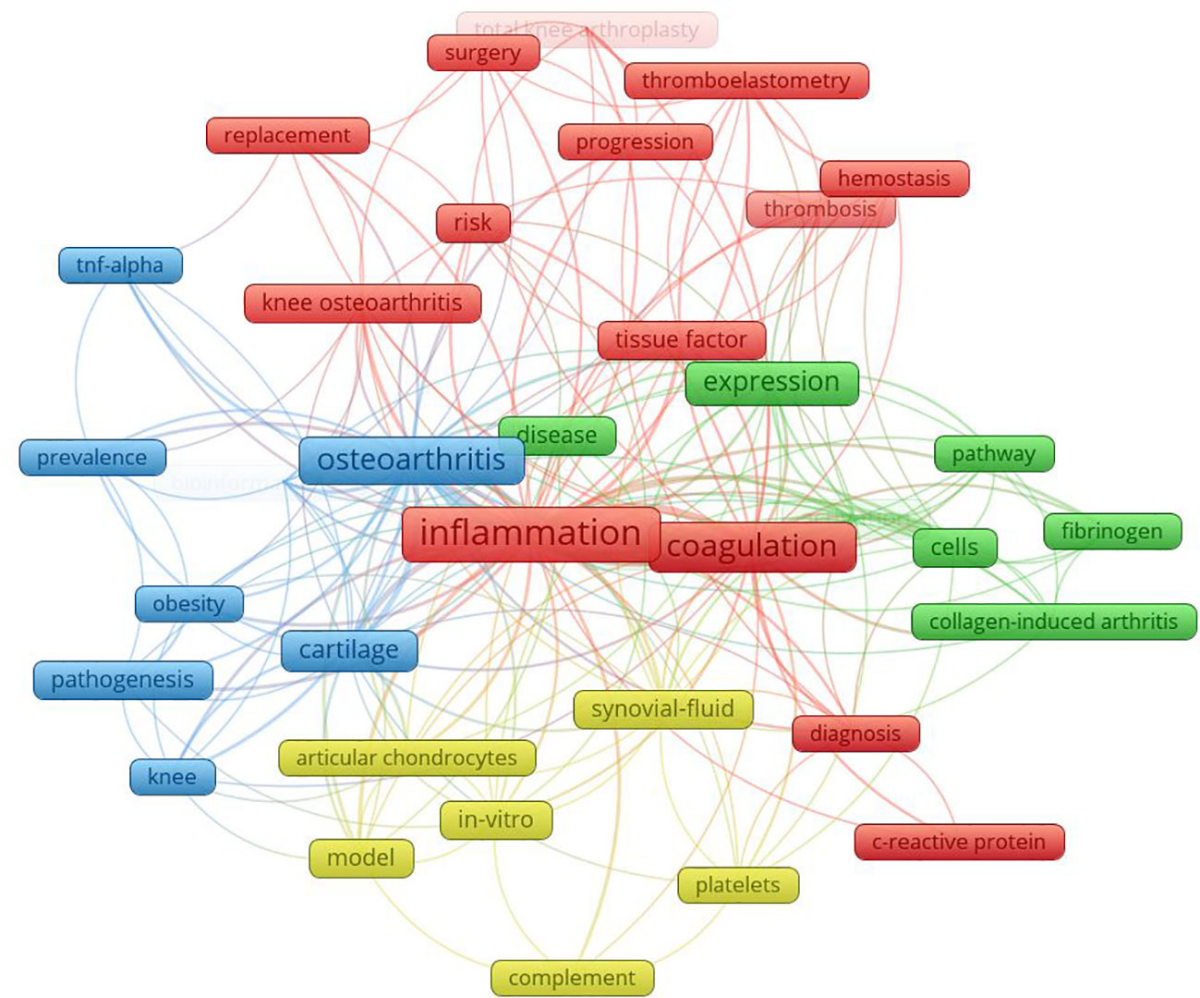
Clustering of bibliometric keywords for hyper-inflammation-associated hypercoagulability in OA. Node size represents the frequency of occurrence, with larger nodes having more occurrences. The thickness of the connections between different nodes reflects the frequency of co-occurrence (the higher the co-occurrence frequency, the thicker the lines), the distance between nodes indicates the degree of closeness of the association (the closer the distance, the closer the connection), the color represents the cluster to which it belongs, nodes of the same color belong to the same research field, and different colors represent different thematic groups.

## 2 Methods

### 2.1 Analysis of keyword co-occurrence network

Based on the preliminary exploratory objectives of this study, keyword co-occurrence network analysis was employed to identify inflammation- and coagulation-related biomarkers. A comprehensive literature search was conducted in the Web of Science Core Collection (WoSCC) on May 10, 2025, using the following search strategy:TS = (“osteoarthritis” OR “OA”) AND TS = (“inflammation”) AND TS = (“coagulation” OR “hypercoagulable”). The search was limited to publications from 2010 to 2025. Two researchers (Fanfan Wang and Yanqiu Sun) independently performed literature screening and data extraction. After reviewing titles, abstracts, and full texts, studies with insufficient relevance to the research topic or informal publications were excluded. In cases of disagreement, a third researcher (Yanyan Fang) was consulted to reach a consensus. Ultimately, 63 articles were included in the analysis. Subsequently, VOSviewer software was used to perform keyword co-occurrence network analysis on the included literature. Keywords with more than three occurrences were selected, and clustering analysis was visualized using VOSviewer.

### 2.2 Patient source

This study was based on the inpatient case data processing system V1.0 (2017SR422234) of the First Affiliated Hospital of Anhui University of Traditional Chinese Medicine. Clinical data from patients with OA were collected in accordance with predefined diagnostic, inclusion, and exclusion criteria. Diagnostic criteria: All patients met the diagnostic criteria for OA proposed by the Chinese Society of Rheumatology in 2010 (26). The diagnostic criteria for TCM syndromes were based on the Guidelines for Diagnosis and Treatment of Osteoarthritis Disease Syndrome Combination (2021) (27). Inclusion criteria (1): Fulfillment of the above diagnostic criteria (2); Availability of complete laboratory indicators before and after treatment. Exclusion criteria (1): Failure to meet the above diagnostic criteria (2); Lack of corresponding laboratory indicators after hospital admission; (3) Inability to effectively communicate or report subjective symptoms; (4) Presence of uncontrolled diseases or other joint system disorders that could significantly affect inflammation, coagulation, or metabolic indicators; (5) Long-term use of high-dose anti-inflammatory drugs or anticoagulant medications. Based on these criteria, data from 30 patients with OA were included in the study. In parallel, 30 healthy individuals were recruited as controls. Peripheral blood samples were collected from both OA patients and healthy controls, and PBMCs were isolated for subsequent analyses. This study was approved by the Ethics Committee of the First Affiliated Hospital of Anhui University of Traditional Chinese Medicine (Ethics No. 2023AH-52).

### 2.3 Clinical data collection of OA patients

The clinical data collected from patients with OA comprised three main components. First, basic demographic and clinical information was obtained, including sex, age, and disease duration. Height and weight were recorded to calculate body mass index (BMI). For enrolled patients with common comorbidities, such as stable hypertension or diabetes, medication histories were documented in detail. These data are summarized in **Supplementary Table 1**. Second, data on inflammatory and coagulation-related indices were collected, including hypersensitive C-reactive protein (hs-CRP), erythrocyte sedimentation rate (ESR), prothrombin time (PT), activated partial thromboplastin time (APTT), platelet count (PLT), thrombin time (TT), D-dimer, fibrinogen (FBG), and international normalized ratio (INR). Finally, the Self-Assessment Scale of Self-Perception of Patients (SPP) with OA was administered. The SPP scale used in this study incorporates TCM syndrome scores and was developed based on a comprehensive evaluation of disease activity, joint function, and anxiety-depression status in patients with OA. This scale enables graded quantification of TCM-related symptoms and signs, providing a standardized and scientifically grounded tool for assessing therapeutic efficacy and guiding clinical treatment and decision-making (28). Having been applied clinically for many years, it reliably reflects changes in self-perception among patients with OA (29, 30). The SPP scale includes the following components: the eight dimensions of the 36-Item Short Form Health Survey (SF-36), namely physical functioning (PF), role limitations due to physical health (RP), bodily pain (BP), general health perceptions (GH), vitality (VT), social functioning (SF), role limitations due to emotional problems (RE), and mental health (MH); the Visual Analogue Scale (VAS); the Self-Rating Anxiety Scale (SAS); the Self-Rating Depression Scale (SDS); and the TCM syndrome score, which includes Cold-Dampness Syndrome (CDS), Dampness-Heat Syndrome (SDH), Blood Stasis Syndrome (SBS), and Dampness Stagnation due to Spleen Deficiency Syndrome (SDSSD). After obtaining informed consent, two trained clinical researchers (Fanfan Wang and Mingyu He) provided standardized guidance to patients on how to complete the questionnaire. Patients were instructed to select the responses that best reflected their current condition. For patients who had difficulty understanding certain items, researchers provided simplified explanations to ensure accurate completion. Upon completion, the questionnaires were collected and entered into a preconfigured data processing system, which automatically calculated and generated scores for each item.

### 2.4 Correlation analysis

Spearman correlation analysis, also known as rank or ordinal correlation, is a non-parametric statistical method used to evaluate the strength and direction of a monotonic relationship between two variables (x, y) (31). The correlation coefficient ranges from -1.0 to 1.0, where -1.0 indicates a perfect negative correlation, 1.0 indicates a perfect positive correlation, and 0.0 indicates no correlation between the variables. Because Spearman correlation does not require the data to follow a normal distribution, it is well-suited for datasets containing non-normal distributions or outliers, as observed in the present study. Accordingly, Spearman correlation analysis was employed to assess the relationship between differentially expressed methyltransferases and lncRNA MEG3 expression in PBMCs from patients with OA. In addition, correlations between the expression of lncRNA MEG3 and KLF4 and clinical indicators, including ESR, hs-CRP, FBG, PT, INR, APTT, TT, D-dimer, and PLT, as well as SPP-related scales (SF-36, VAS, SAS, SDS, CDS, SDH, SDSSD, and SBS) were further analyzed.

### 2.5 Analysis of association rules

Association rule analysis is a data mining technique used to uncover relationships among variables, with the Apriori algorithm being the most widely applied approach (32). The effectiveness of association rules is primarily evaluated using three metrics: support, confidence, and lift. Support represents the probability that two items occur together within a dataset, while confidence reflects the strength of the association between them. Lift indicates the degree of dependency between two variables: a lift value of 1 indicates no association, a value less than 1 indicates a negative association with mutual exclusivity, and a value greater than 1 indicates a positive association, suggesting that the variables are likely to co-occur. These metrics collectively determine whether an association rule is meaningful and worth retaining. Typically, minimum thresholds for support and confidence are predefined, and only rules meeting these criteria are considered strongly associated (33). In this study, the Apriori algorithm was applied to perform association rule analysis. Based on changes in clinical indicators of OA patients before and after treatment, indicator values were binarized, with “indicator improvement” assigned a value of 1 and “no improvement or no change” assigned a value of 0. The minimum support and confidence thresholds were set at 50% and 70%, respectively, and the maximum number of antecedent items was limited to one (34, 35). IBM SPSS Modeler version 18.0 was used to identify Chinese herbal compound preparations that were highly correlated with improvements in clinical indicators among OA patients, and the results were visualized and analyzed. Considering the potential risk of overfitting when applying relatively high support and confidence thresholds in association rule mining with a small sample size, a sensitivity analysis was further conducted. The support and confidence thresholds were gradually reduced to more conservative levels (support set at 30% and confidence at 40%). Based on the newly generated rule set, the stability of the previously identified core rules was evaluated to determine whether they consistently appeared under these more lenient conditions.

### 2.6 Prediction of m^6^A modification of lncRNA MEG3

The gene sequence of lncRNA MEG3 was retrieved from the NCBI Gene database (https://www.ncbi.nlm.nih.gov/gene) and subsequently imported into the deepSRAMP platform (http://www.cuilab.cn/deepsramp/) for prediction of potential m6A modification sites. In addition, the RM2Target online database was used to predict the potential m6A “writers” and “erasers” that bind to lncRNA MEG3.

### 2.7 RNA pull -down validation of lncRNA MEG3 binding proteins

Sense and antisense PCR primers for lncRNA MEG3 were designed and synthesized. A total of 2×10^7^ cells were collected, and whole-cell proteins were extracted. Nucleic acids were removed from the protein samples, and the supernatant was collected. A 100 μL aliquot of the supernatant was retained as the input group, while the remaining supernatant was divided equally into two tubes (0.8 mL per tube), labeled as RNA pull-down (RPD) and negative control (NC) samples, respectively, and stored at -80°C for subsequent use. According to a length-to-mass ratio of 1 μg per 1,000 nucleotides, appropriate amounts of biotin-labeled RNA probes and NC probes were prepared and added to 50 μL RNA structure buffer and 20 μL RNase-free water to allow formation of RNA secondary structures. For probe magnetic bead preparation, 40 μL streptavidin magnetic beads were transferred into two RNase-free centrifuge tubes. The beads were washed with 1 mL 1× TES buffer, allowed to stand for 1 min, and the washing solution was removed. Subsequently, approximately 100 μL of RNA probes with formed secondary structures, 100 μ L RNase-free water, and 200 μL 2× TES buffer were added to the beads. After incubation and removal of the supernatant, 0.5 mL 1× TES buffer was added to each tube. The beads were gently rotated for washing and allowed to stand before removing the supernatant. This washing procedure was repeated twice. The probe-bead complexes were then incubated with cell extracts, followed by the addition of 5 μL RNase inhibitor and 5 μL yeast tRNA. The mixture was gently rotated and incubated at room temperature (25°C) for 2h to allow binding. Magnetic beads were subsequently collected and washed four times with NT2 buffer. RPD and NC beads were then incubated with 60 μ L protein elution buffer and 0.6 μL dithiothreitol (DTT). Eluted proteins were subjected to silver staining using a rapid silver staining kit to detect differential protein bands. Finally, Western blot (WB) analysis was performed to verify the binding relationship between MEG3 and KLF4.

### 2.8 Preparation of drug-containing serum

Thirty clean-grade male Sprague-Dawley (SD) rats (aged 6–8 weeks, weighing 200 ± 20 g; Pizhou Oriental Breeding Co., Ltd., Jiangsu, China) were fed in the standard clean laboratory animal room of the First Affiliated Hospital of Anhui University of Traditional Chinese Medicine under a quiet indoor environment with a uniform lighting, temperature of 18–22°C and a relative humidity of 50–75%. Ventilation and exhaustion were maintained at 10–20 times per hour. After a week of acclimatization, the 30 rats were randomly divided into the XFC drug-containing serum group (20 rats) and the blank serum group (10 rats). The rats in the drug-containing serum group were administered with XFC suspension by gavage at 2 ml/100g (6.48 g/kg/d, 20 times the clinical equivalent dose). The 10 rats in the blank serum group were given the same volume of physiological saline. Before administration, all rats were fasted for 12 hours and allowed to drink water freely to control the impact of food on drug absorption. The treatment was administered once daily for 7 consecutive days. Two hours after the last administration, the rats were anesthetized with sodium pentobarbital (30 mg/kg) and blood was collected from the abdominal aorta. The serum was collected after centrifugation at 3000 r/min for 15 minutes, and then immediately heat-inactivated at 56°C for 30 minutes to eliminate complement interference. The serum was then aliquoted, labeled, and stored at -80°C in a refrigerator for future use, avoiding repeated freeze-thaw cycles. This study was reviewed and ratified by the Laboratory Animal Ethics Committee of Anhui University of Traditional Chinese Medicine (AHUCM-rats-2024068).

### 2.9 Construction and grouping of the co-culture model

CHs were routinely digested and resuspended in complete culture medium. OA-derived PBMCs (OA-PBMCs) and CHs were co-cultured using a Transwell system at a ratio of 2.5:1, with OA-PBMCs seeded in the upper chamber and CHs seeded in the lower chamber. After 48h of co-culture, subsequent experiments were performed. The experimental groups were established as follows: CHs group, Model group (OA-PBMCs+CHs), and Model+JHTL group.

### 2.10 Cell counting kit-8 assay for evaluating the effects of XFC -containing serum on the viability of co-cultured CHs

Cells were seeded into culture plates and incubated overnight at 37°C in a humidified atmosphere containing 5% CO_2_. The CCK-8 assay was used to evaluate the effects of OA-PBMCs on CH viability at different co-culture ratios (0:1, 10:1, 5:1, 2.5:1, and 1:1) and incubation times (12 h, 24 h, and 48 h) to determine the optimal ratio and duration of interaction between OA-PBMCs and CHs. Subsequently, XFC-containing serum at different concentrations (5%, 10%, 20%, 30%, and 40%) was added to the co-cultured cells. After incubation for different time periods (24 h, 48 h, and 72 h), CH viability was assessed using the CCK-8 assay to determine the optimal concentration and treatment duration of XFC -containing serum.

### 2.11 Colorimetric detection of total m^6^A level

Total m^6^A levels were measured using the EpiQuik™ m^6^A RNA Methylation Quantification Kit. RNA samples were added to wells containing binding solution and incubated for 90 min, followed by washing steps. Subsequently, the capture antibody, detection antibody, and enhancement solution were sequentially diluted and added, with incubation times of 60 min, 30 min, and 30 min, respectively. After each incubation, the wells were washed thoroughly. Finally, a color-developing solution was added to monitor color changes, the reaction was terminated with a stop solution, and absorbance was measured at 450 nm.

### 2.12 Methylated RNA immunoprecipitation quantitative polymerase chain reaction

Total RNA was extracted using TRIzol reagent. MeRIP magnetic beads were incubated with anti-m^6^A antibodies, followed by the addition of immunocapture buffer and washing steps to remove unbound antibodies. Subsequently, 1 μg of RNA was added to 20 μL immunocapture buffer, followed by 1.5 μL of negative DNA extraction solution and 1 μL of cleavage enzyme mix, and incubated at room temperature. Next, 20 μL of RNA purification solution was added to each sample and negative control tube, followed by the addition of 160 μL anhydrous ethanol to facilitate RNA release and recovery. Reverse transcription was performed using random primers, and quantitative PCR was conducted using primers targeting regions near the m6A modification site of lncRNA MEG3. Cycle threshold (Ct) values were recorded. The primer sequences for lncRNA MEG3 were as follows: Forward primer (5 ‘→ 3’): AGGATGAAGGACCGGAAC, Reverse primer (5 ‘→ 3’): CCCTCACTAGGGCATTGGTT. It should be noted that, due to the limited availability of clinical tissue samples, m^6^A epitranscriptomic analyses, including total m^6^A level measurement and MeRIP-qPCR, were performed on a subset of samples (n = 3 randomly selected from each group) for exploratory analysis.

### 2.13 Reverse transcription quantitative polymerase chain reaction

Total RNA was extracted from OA-CHs in each group using TRIzol reagent. Reverse transcription was performed to synthesize cDNA, followed by PCR amplification and agarose gel electrophoresis. Semi-quantitative analysis of PCR products was conducted using the relative quantification method, with β-actin serving as the internal reference gene. Relative gene expression levels were calculated using the 2^-△△Ct^ method. Primer sequences for the target genes are listed in **Table 1**:

**Table 1:** Gene primer information.

Target	Primer sequence (5′–3′)	Amplicon Size(bp)
β-actin	Forward:5-'CCCTGGAGAAGAGCTACGAG-3'	96
	Reverse:5-'GGAAGGAAGGCTGGAAGAGT-3'	
LncMEG3	Forward:5-'AGGATGAAGAGGACCGGAAC-3'	75
	Reverse:5-'CCCTCACTAGGGCATTGGTT-3'	
METTL3	Forward:5-'GCCTTCTGAACCAACAGTCC-3'	184
	Reverse: 5-'CTGGCTTTCATGCACTCCTC-3'	
METTL14	Forward:5-'TGCCTGTGATGGGTCCTTAG-3'	132
	Reverse: 5-'ACAGGTGCCTATGCCATGTA-3'	
WTAP	Forward:5-'AAGCAACAACAGCAGGAGTC-3'	163
	Reverse: 5-'TCGCTGGGTCTACCATTGTT-3'	

### 2.14 WB analysis

Total cellular protein was extracted using 100 μL of RIPA lysis buffer, and 5× SDS-PAGE loading buffer was added at a ratio of 1:4. The samples were heated in a boiling water bath for 10 min to ensure complete protein denaturation. After cooling to room temperature, proteins were separated by SDS-PAGE and transferred onto membranes. The membranes were blocked with WB blocking solution containing 5% skim milk powder at room temperature for 2 h. Primary antibodies were diluted according to the following ratios: CBLL1 (1:500), KIAA1429 (1:2000), METTL14 (1:500), METTL16 (1:500), METTL3 (1:500), RBM15B (1:2000), WTAP (1:2000), ZC3H13 (1:500), ALKBH5 (1:2000), FTO (1:2000), β-actin (1:1000), and KLF4 (1:500). After incubation with primary antibodies and subsequent membrane washing, horseradish peroxidase (HRP)-conjugated secondary antibodies, goat anti-mouse IgG (1:10,000) and goat anti-rabbit IgG (1:10,000), were applied. Following membrane washing, an enhanced chemiluminescence (ECL) substrate was added for signal development. Excess liquid was removed, and protein bands were visualized by adjusting exposure conditions according to signal intensity. Band intensities were quantified using ImageJ software.

### 2.15 Enzyme-linked immunosorbent assay

Commercial ELISA kits were used to measure the concentrations of inflammatory and coagulation-related factors in the supernatants of co-cultured CHs and PBMCs. The following kits were employed: IL-6 ELISA kit (Cat. No. RX106126H), IL-10 ELISA kit (Cat. No. RX103064H), TNF-α ELISA kit (Cat. No. RX104793H), TF ELISA kit (Cat. No. RX104729H), plasminogen activator inhibitor-1 (PAI-1) ELISA kit (Cat. No. RX105072H), and prostaglandin I_2_ (PGI_2_) ELISA kit (Cat. No. RX105371H). All procedures were performed in accordance with the manufacturers’ instructions.

### 2.16 Statistical methods

Clinical data were analyzed using SPSS version 23.0 (IBM, Armonk, NY, USA), and experimental data were analyzed using GraphPad Prism (version 9.0.0). Continuous variables with a normal distribution are presented as mean ± standard deviation (SD), whereas variables with a non-normal distribution are expressed as median (interquartile range), M (P25, P75). Comparisons between two groups were performed using unpaired t-tests. Comparisons among multiple groups were conducted using one-way analysis of variance (ANOVA) or repeated-measures ANOVA, with Bonferroni correction applied for multiple comparisons. A *p* value < 0.05 was considered statistically significant.

## 3 Results

### 3.1 Co-occurrence network of keywords related to hyperinflammation-associated hypercoagulability in OA

Given the complexity of the link between hyperinflammation and hypercoagulability, we first employed bibliometric big-data analysis to determine whether osteoarthritis research exhibits molecular clusters dominated by inflammation, coagulation, or their cross-regulation. This approach also aimed to identify the most central and highly investigated molecular nodes, thereby providing priority directions and focal points for subsequent in-depth mechanistic studies. According to bibliometric theory, keyword co-occurrence network analysis offers reproducible and comparable quantitative indicators that reflect research hotspots and trends within a specific field. Therefore, analysis of key indicators is essential (36). In a keyword co-occurrence network, node size is generally proportional to the frequency of keyword occurrence. Nodes are connected by lines, the thickness of which represents the frequency of co-occurrence, with thicker lines indicating stronger associations. The distance between nodes reflects the closeness of their relationships, with shorter distances indicating stronger correlations. Different colors represent distinct clusters, with nodes of the same color typically belonging to the same research theme. Collectively, these features provide an overview of the core research focus on hyperinflammation-associated hypercoagulability in OA.

As shown in **Figure 1**, four major clusters were identified around the central themes of OA, inflammatory responses, and hypercoagulable states, representing four main research directions. The red cluster primarily involved the inflammatory marker CRP and hypercoagulability-related keywords such as tissue factor and thrombus formation. The blue cluster was associated with OA pathogenesis and epidemiology, including TNF-α and obesity. The yellow cluster mainly comprised keywords related to cellular models used in OA research. The green cluster focused on signaling pathways and extracellular matrix-related components, including collagen type I and FBG. Based on the above analysis of key indicators, TNF-α, IL-6, TF, and PAI-1 were selected as focal biomarkers in the study of hyperinflammation-associated hypercoagulability in OA. In addition, in combination with previous studies, the anti-inflammatory factor IL-10 and the anticoagulant factor PGI_2_ were selected as reference indicators to evaluate changes in pro-inflammatory and procoagulant factors.

**Figure 2:**
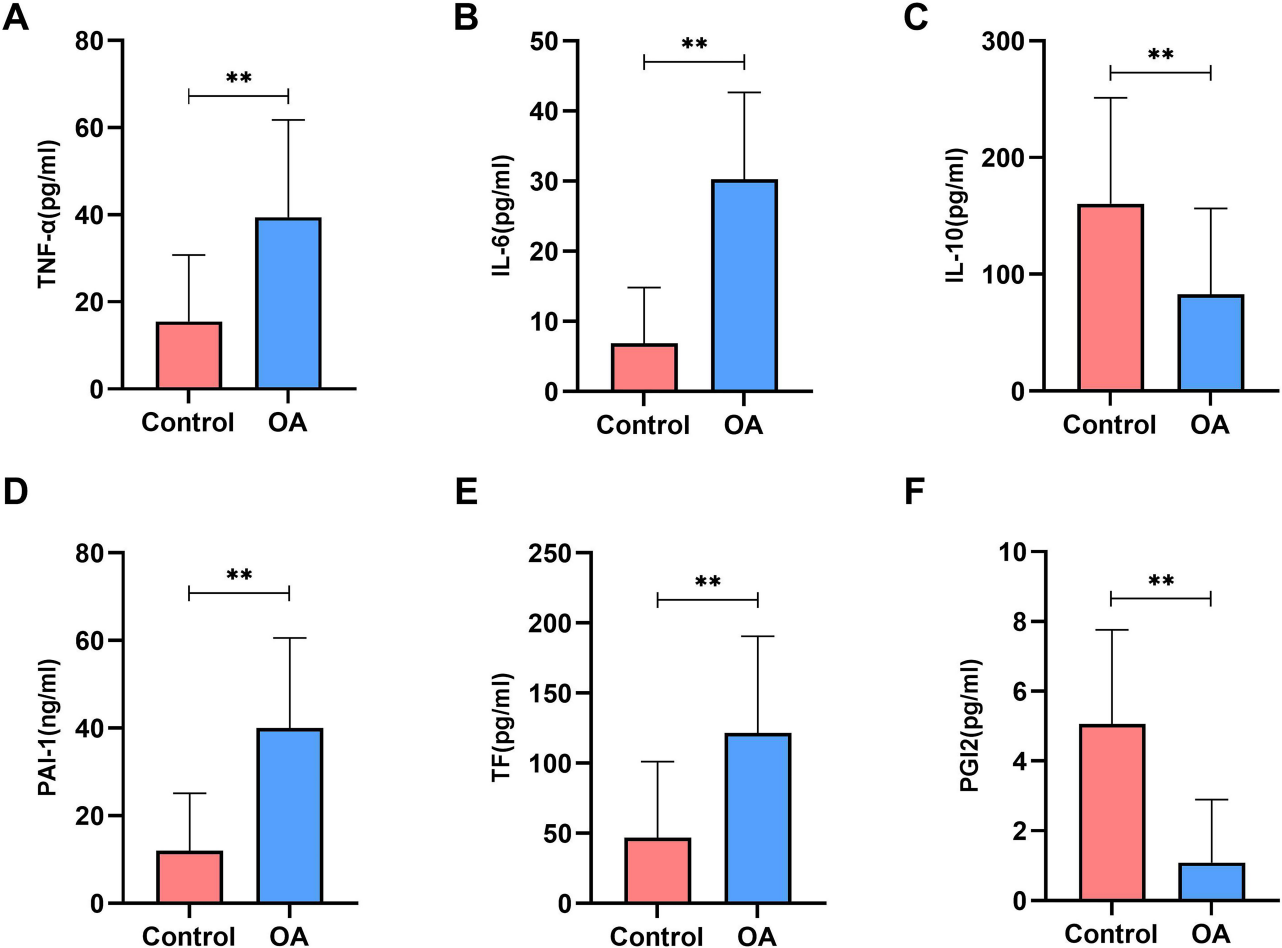
Expression levels of inflammation and coagulation factors in PBMCs of healthy controls and OA patients. **(A)** ELISA detection of TNF-α; **(B)** ELISA detection of IL-6; **(C)** ELISA detection of IL-10; **(D)** ELISA detection of PAI-1; **(E)** TF; **(F)** ELISA detection of PGI2. n=30, **P<0.001.

### 3.2 OA patients exhibit hyperinflammation-associated hypercoagulability and abnormal SPP

The keyword co-occurrence network analysis identified key focal points in the field of inflammation-associated hypercoagulability in osteoarthritis, suggesting a potential association between inflammatory and coagulation-related factors in OA. However, this association requires experimental validation to support causal inference. Therefore, peripheral blood samples were collected from 30 patients with OA and 30 healthy individuals to measure the selected cytokines and coagulation-related factors. The results showed that, compared with healthy controls, the expression levels of the pro-inflammatory cytokines TNF-α and IL-6 in PBMCs from patients with OA were significantly increased (*p* < 0.01), whereas the expression level of the anti-inflammatory cytokine IL-10 was significantly decreased (*p* < 0.01). In addition, the levels of the procoagulant factors PAI-1 and TF were significantly elevated (*p* < 0.01), while the level of the anticoagulant factor PGI_2_ was significantly reduced (*p* < 0.01) in OA patients (**Figures 2A-F**).

**Figure 3:**
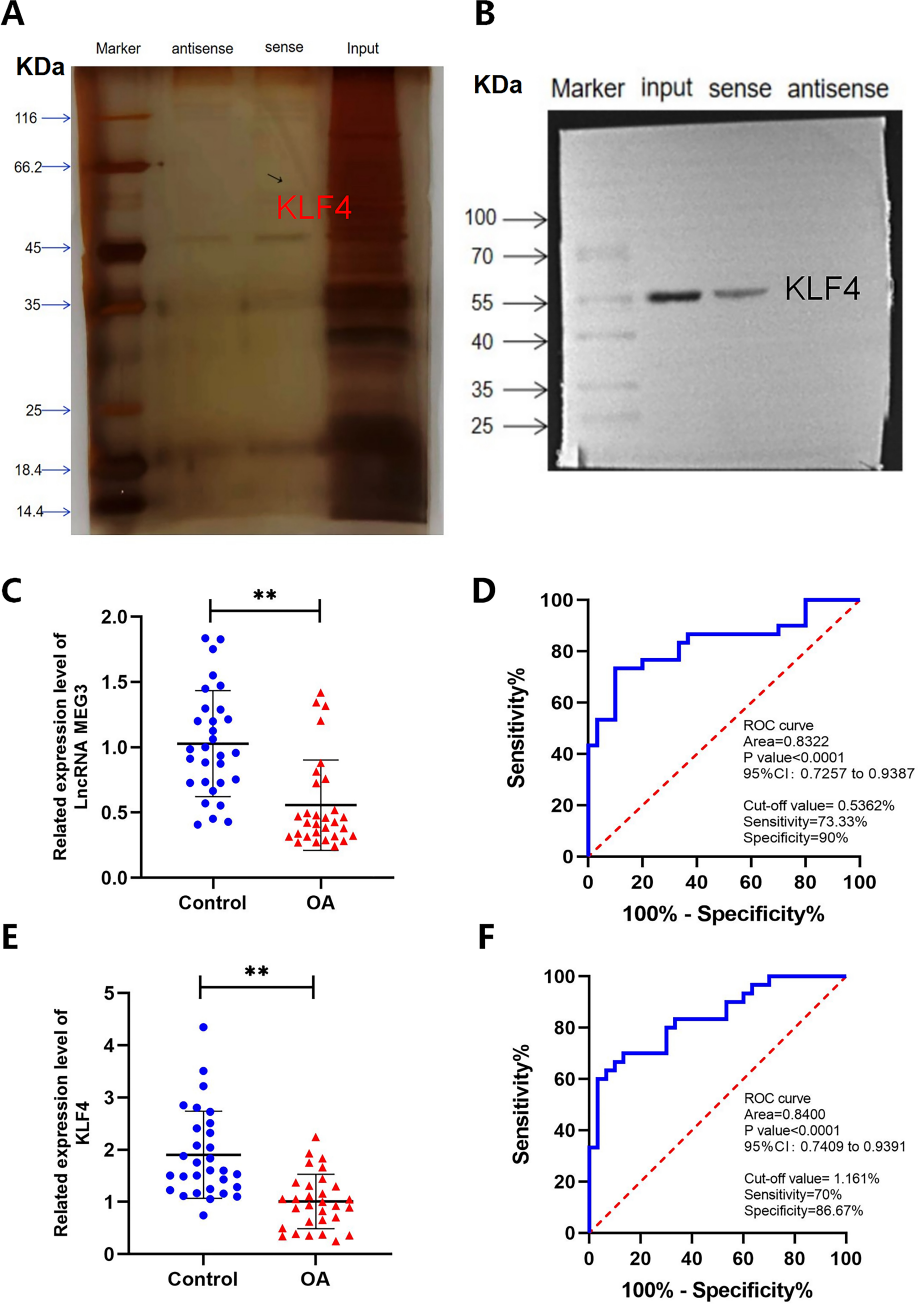
Binding and expression of LncRNA MEG3 and KLF4. **(A, B)** Silver staining gel image and WB validation of LncRNA MEG3 targeting KLF4. **(C)** The expression levels of lncRNA MEG3 in PBMCs of healthy controls and OA patients; **(D)** ROC curve analysis of lncRNA MEG3; **(E)** The expression levels of KLF4 in PBMCs of healthy controls and OA patients; **(F)** ROC curve analysis of KLF4. n=30. **P < 0.001.

Further analysis of clinical laboratory indicators revealed that inflammatory markers, including ESR and hs-CRP, were significantly increased in patients with OA. Coagulation-related indicators, such as PLT, FBG, and D-dimer, were also significantly elevated, indicating the presence of pronounced inflammatory responses and a hypercoagulable state in OA patients (**Table 2**). Analysis of the SPP scale demonstrated that scores across all eight dimensions of the SF-36 were significantly reduced, whereas scores for the SAS, SDS, and disease activity assessed by the VAS were significantly increased. Notably, TCM syndrome assessment showed that scores for CDS, SDH, SBS, and SDSSD were elevated to varying degrees. These findings indicate that patients with active OA exhibit abnormal self-perception, which severely impairs their quality of life. Collectively, these results highlight the need for further clarification of OA pathogenesis and underscore the importance of identifying effective therapeutic agents to improve hyperinflammation-associated hypercoagulability and SPP abnormalities in patients with OA.

**Table 2:** The changes in indicators of patients with osteoarthritis.

Index	OA (n=30)	[Min, Max]/Normal reference range
Inflammatory indicators		
ESR(mm/h)	49.30(39.25, 60.00)	0-38mm/h
hs-CRP(mg/L)	23.91(4.71, 49.84)	<1mg/L
Coagulation indicators		
PLT(×10^9/L)	200.00(165.50, 223.00)	100×10^9/L-300×10^9/L
FBG(g/L)	3.94(2.58, 4.44)	2-4g/L
INR	0.95(0.86, 1.02)	0.89-1.25
APTT(sec)	27.44(26.28, 29.30)	25-33.8
TT(sec)	19.06(17.90, 19.93)	14-21
PT(sec)	10.81(10.00, 10.98)	9-13
D-Dimer(mg/L)	1.37(0.23, 1.81)	0-0.55
VAS(score)	3.93(3.00, 5.00)	0
SAS(score)	44.43(40.94, 50.31)	<50
SDS(score)	52.13(44.69, 59.06)	<50
SF-36(score)		
PF	52.00(25.00, 85.00)	>50
RP	20.33(0.00, 25.00)	>50
BP	31.52(29.43, 31.00)	>50
GH	35.87(30.00, 45.00)	>50
VT	41.83(30.00, 55.00)	>50
SF	49.82(37.50, 62.50)	>50
RE	38.33(0.00, 66.67)	>50
MH	48.98(33.00, 60.00)	>50
TCM Syndrome Score(score)		
CDS	9.10(6.00, 14.00)	NA
SDH	9.47(6.00, 11.25)	NA
SDSSD	10.07 (9.00, 11.25)	NA
SBS	6.90(4.75, 9.25)	NA

### 3.3 LncRNA MEG3 and KLF4 are closely related to hyperinflammation-associated hypercoagulability in OA

**Figure 4:**
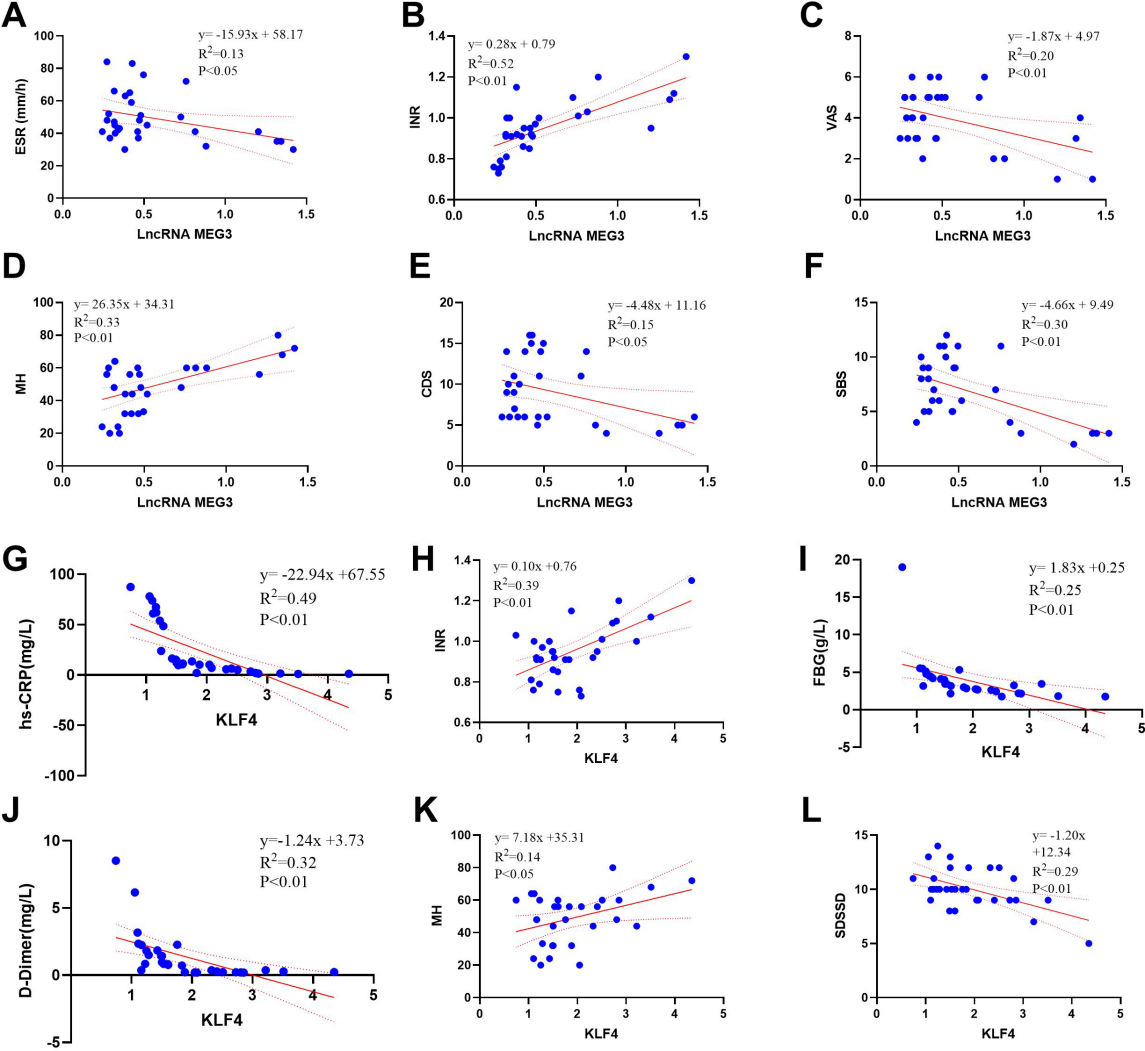
Correlation analysis of LncRNA MEG3 and KLF4 with laboratory indicators and SPP in OA patients. **(A–F)** Correlation analysis between LncRNA MEG3 and laboratory indicators and SPP in OA patients; **(G–L)** Correlation analysis between KLF4 and laboratory indicators and SPP in OA patients. n=30.

Previous studies have demonstrated that downregulation of lncRNA MEG3 contributes to inflammation-associated hypercoagulability in patients with OA (16). However, the underlying regulatory mechanisms remain to be further elucidated. To address this, RPD assays were performed to identify downstream target genes of lncRNA MEG3, leading to the identification of KLF4 by mass spectrometry analysis (**Figures 3A, B**). Subsequently, the expression levels of lncRNA MEG3 and KLF4 in OA patients were examined, and their associations with clinical indicators and SPP were analyzed using peripheral blood samples from patients with OA and healthy controls. The results showed that lncRNA MEG3 expression was significantly reduced in PBMCs from patients with OA, with an area under the receiver operating characteristic (ROC) curve of 0.8322 (**Figures 3C, D**). Similarly, KLF4 expression was significantly decreased in PBMCs from OA patients, with an area under the ROC curve of 0.8400 (**Figures 3E, F**). Correlation analysis further revealed that lncRNA MEG3 expression in OA patients was negatively correlated with ESR, VAS, CDS, and SBS scores, while showing positive correlations with INR and the MH dimension of the SF-36. In addition, KLF4 expression was negatively correlated with hs-CRP, D-dimer, FBG, and SDSSD, and positively correlated with INR and MH (**Figures 4A–L**).

**Figure 5:**
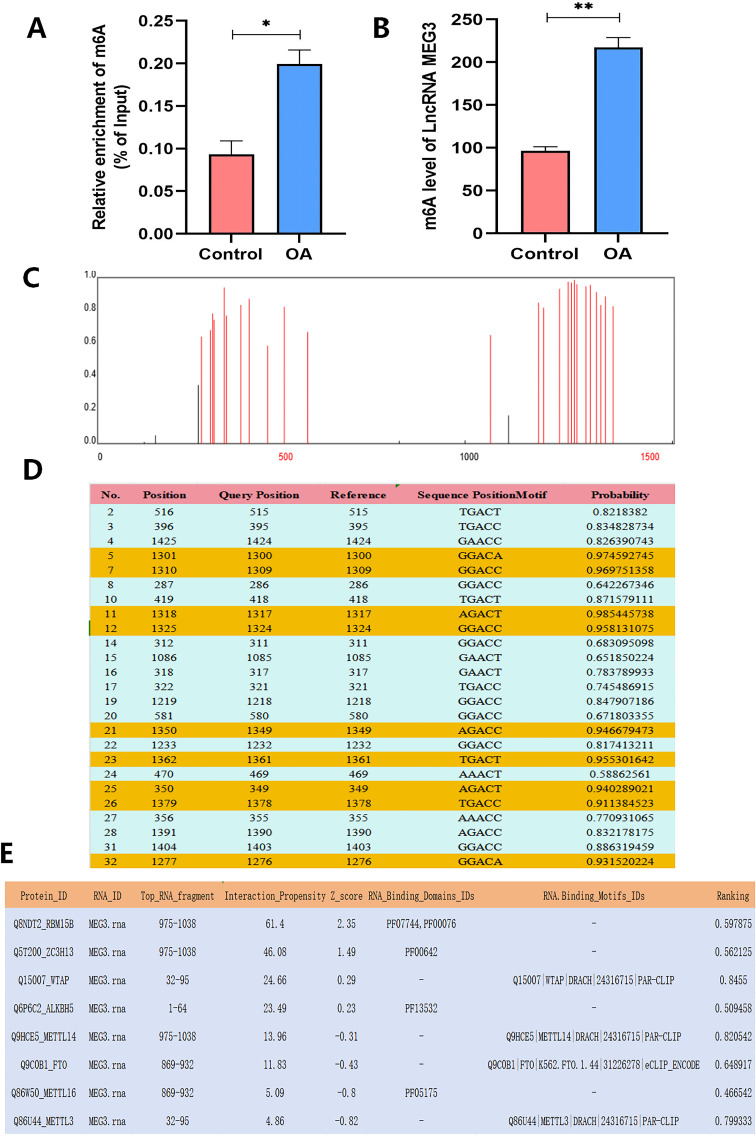
Prediction of m6A methylation sites in LncRNA MEG3. **(A)** Total m6A level detected by colorimetric method; **(B)** MeRIP-qPCR was used to detect the level of LncRNA MEG3 m6A; **(C, D)** SRAMP software predicts the methylation site of LncRNA MEG3; **(E)** RM2 Target predicts the binding of LncRNA MEG3 to methylases and demethylases. n=3, **P < 0.001,*P < 0.05.

### 3.4 LncRNA MEG3 undergoes m^6^A modification

PBMCs from three patients with OA and three healthy controls were selected for preliminary analysis to evaluate total m^6^A levels and m^6^A modification of lncRNA MEG3. The results demonstrated that both global m^6^A levels and lncRNA MEG3 m^6^A levels showed an increasing trend in OA patients compared with healthy controls, suggesting the presence of abnormal m6A modification in OA. These findings imply that dysregulated m^6^A modification may contribute to OA pathogenesis by influencing lncRNA MEG3 expression (**Figures 5A, B**). Based on these observations, five potential m^6^A modification sites with greater than 95% confidence were predicted in the lncRNA MEG3 sequence using the deepSRAMP database (http://www.cuilab.cn/deepsramp/). These high-confidence sites are highlighted in bold yellow within the sequence (**Figures 5C, D**). Subsequently, upstream m^6^A-related regulatory enzymes of lncRNA MEG3 were predicted using the RM2Target online platform (http://rm2target.canceromics.org/). The analysis indicated that the methyltransferases METTL3, METTL14, WTAP, RBM15/15B, ZC3H13, and METTL16, as well as the demethylases FTO and ALKBH5, exhibited strong binding potential with lncRNA MEG3 (**Figure 5E**).

**Figure 6:**
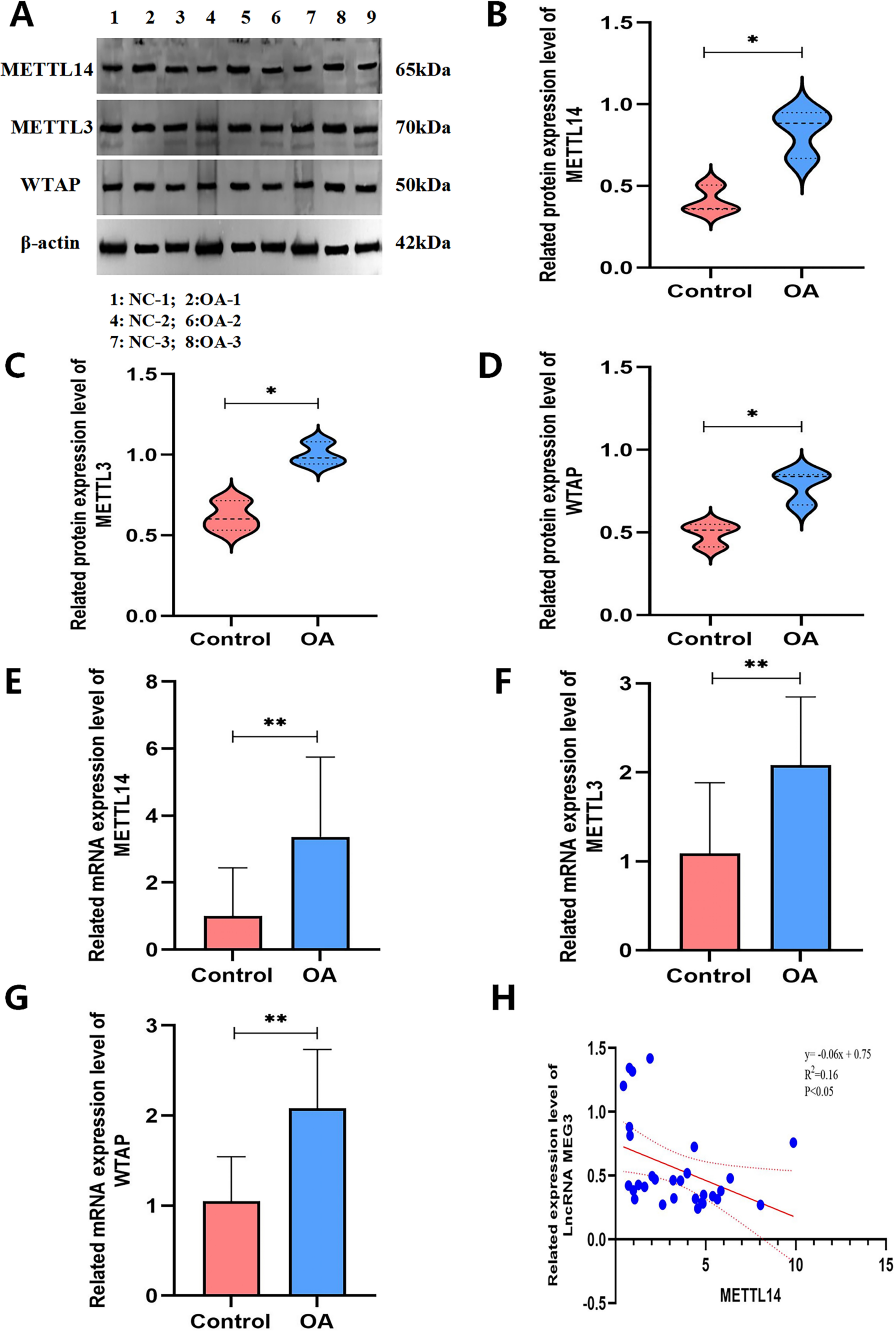
Methyltransferase METTL14 may mediate LncRNA MEG3 m6A modification. **(A–D)** Methylase levels detected by WB for differential expression (n=3); **(E–G)** RT-qPCR detection of differentially expressed methyltransferase levels (n=30); **(H)**: Correlation analysis between differentially expressed methyltransferase METTL14 and Lnc RNA MEG3 expression levels (n=30). **P < 0.001,*P < 0.05.

### 3.5 Methyltransferase METTL14 may mediate m^6^A modification of lncRNA MEG3

To validate the database prediction results, the expression levels of m6A methyltransferases (METTL3, METTL14, WTAP, RBM15/15B, ZC3H13, and METTL16) and demethylases (FTO and ALKBH5) were examined in PBMCs from three patients with OA and three healthy controls. WB analysis revealed significant differential expression of the methyltransferases METTL3, METTL14, and WTAP between the two groups (*p* < 0.05) (**Figures 6A, D**). Subsequently, the sample size was expanded to further assess the expression of METTL3, METTL14, and WTAP in PBMCs from 30 patients with OA and 30 healthy controls, and to clarify their correlations with lncRNA MEG3 expression. The results demonstrated that METTL14 expression was significantly elevated in PBMCs from OA patients and was strongly correlated with the downregulation of lncRNA MEG3 expression (*p* < 0.001) (**Figures 6E–H**).

**Figure 7:**
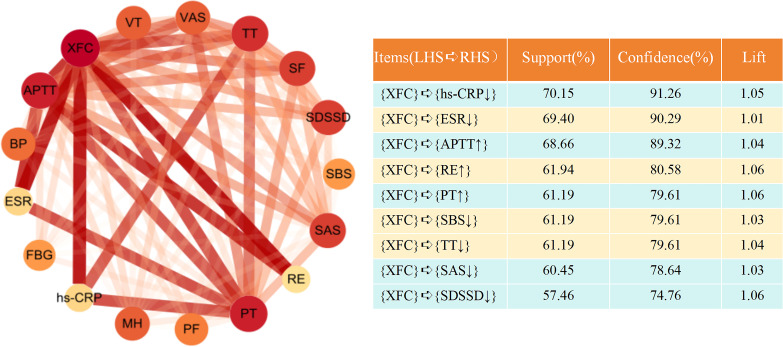
Association rule network diagram of XFC and improvement of inflammation, coagulation indicators, and SPP scores in OA patients. Node size represents support, with higher support indicating larger nodes; The thickness of the line represents confidence, and the higher the confidence, the thicker the line; Color represents the degree of improvement, and the higher the degree of improvement, the brighter the color.

### 3.6 XFC is a key therapeutic agent for improving hyperinflammation-associated hypercoagulability and SPP in OA patients

To further identify therapeutic agents capable of improving the hyperinflammatory and hypercoagulable state in OA, changes in clinical indicators of OA patients before and after treatment were first analyzed. The results demonstrated that, compared with baseline values, SPP-related scores, including VAS, SAS, SDS, RP, BP, GH, VT, SF, RE, MH, CDS, SDH, SDSSD, and SBS, were significantly improved after treatment (*p* < 0.01). In addition, inflammatory indicators (hs- CRP and ESR) and coagulation-related indicators (TT, FBG, and D-dimer) also showed significant improvement following treatment (*p* < 0.01) (**Table 3**).

**Table 3:** Changes in clinical indicators and SPP of OA patients before and after treatment.

Index	Pre-treatment	Post-treatment	Z-value	P-value
ESR(mm/h)	49.30(39.25, 60.00)	14.27(3.50, 20.75)	-4.70	<0.01
hs-CRP(mg/L)	23.91(4.71, 49.84)	3.18(1.06, 5.02)	-4.08	<0.01
PLT(×10^9/L)	200.00(165.50, 223.00)	208.50(159.50, 242.50)	-0.57	0.57
FBG(g/L)	3.94(2.58,4.44)	2.91(2.50, 3.27)	-2.17	0.03
INR	0.95(0.86,1.02)	0.97(0.90, 1.09)	-0.53	0.60
APTT(sec)	27.44(26.28, 29.30)	29.26(27.93, 30.60)	-1.83	0.36
TT(sec)	19.06(17.90, 19.93)	17.46(16.80, 18.67)	-3.26	<0.01
PT(sec)	10.81(10.00, 10.98)	10.87(10.00,11.80)	-0.96	0.34
D-Dimer(mg/L)	1.37(0.23, 1.81)	0.40(0.22,0.56)	-3.15	<0.01
VAS	3.93(3.00, 5.00)	2.72(2.00, 3.05)	-2.75	<0.01
SAS	44.43(40.94, 50.31)	38.28(36.25, 40.00)	-3.74	<0.01
SDS	52.13(44.69, 59.06)	41.74(37.50, 45.00)	-3.85	<0.01
PF	52.00(25.00, 85.00)	62.50(43.75, 90.00)	-1.17	0.24
RP	20.33(0.00, 25.00)	56.60(50.00, 75.00)	-3.60	<0.01
BP	31.52(29.43, 31.00)	52.63(41.00, 61.99)	-4.40	<0.01
GH	35.87(30.00, 45.00)	47.07(43.75, 56.99)	-3.09	<0.01
VT	41.83(30.00, 55.00)	63.17(55.00, 70.00)	-4.02	<0.01
SF	49.82(37.50, 62.50)	68.75(62.50, 75.00)	-4.00	<0.01
RE	38.33(0.00, 66.67)	64.84(61.25, 66.78)	-3.27	<0.01
MH	48.98(33.00, 60.00)	67.33(63.00, 76.00)	-3.68	<0.01
CDS	4.93(3.75, 6.25)	6.00(4.00, 8.00)	-4.14	<0.01
SDH	3.53(2.00, 4.00)	7.00(6.00, 9.00)	-4.68	<0.01
SBS	3.33(2.00, 4.00)	3.00(2.00, 5.00)	-4.39	<0.01
SDSSD	5.20(3.00, 8.00)	6.00(4.00, 8.00)	-4.36	<0.01
SDSSD	5.20(3.00, 8.00)	6.00(4.00, 8.00)	-4.36	<0.01

Subsequently, association rule analysis was performed to elucidate the relationship between pharmacological interventions and improvements in clinical indicators. The resulting association network revealed that XFC was significantly associated with reductions in hs-CRP, ESR, PLT, SAS, SDS, SDSSD, and SBS, and showed strong associations with increases in APTT, RE, and PT (**Figure 7**, **Supplementary Table 2**). These findings indicate that XFC effectively alleviates inflammatory responses, improves hypercoagulability, and enhances self-perception in patients with OA.

**Figure 8:**
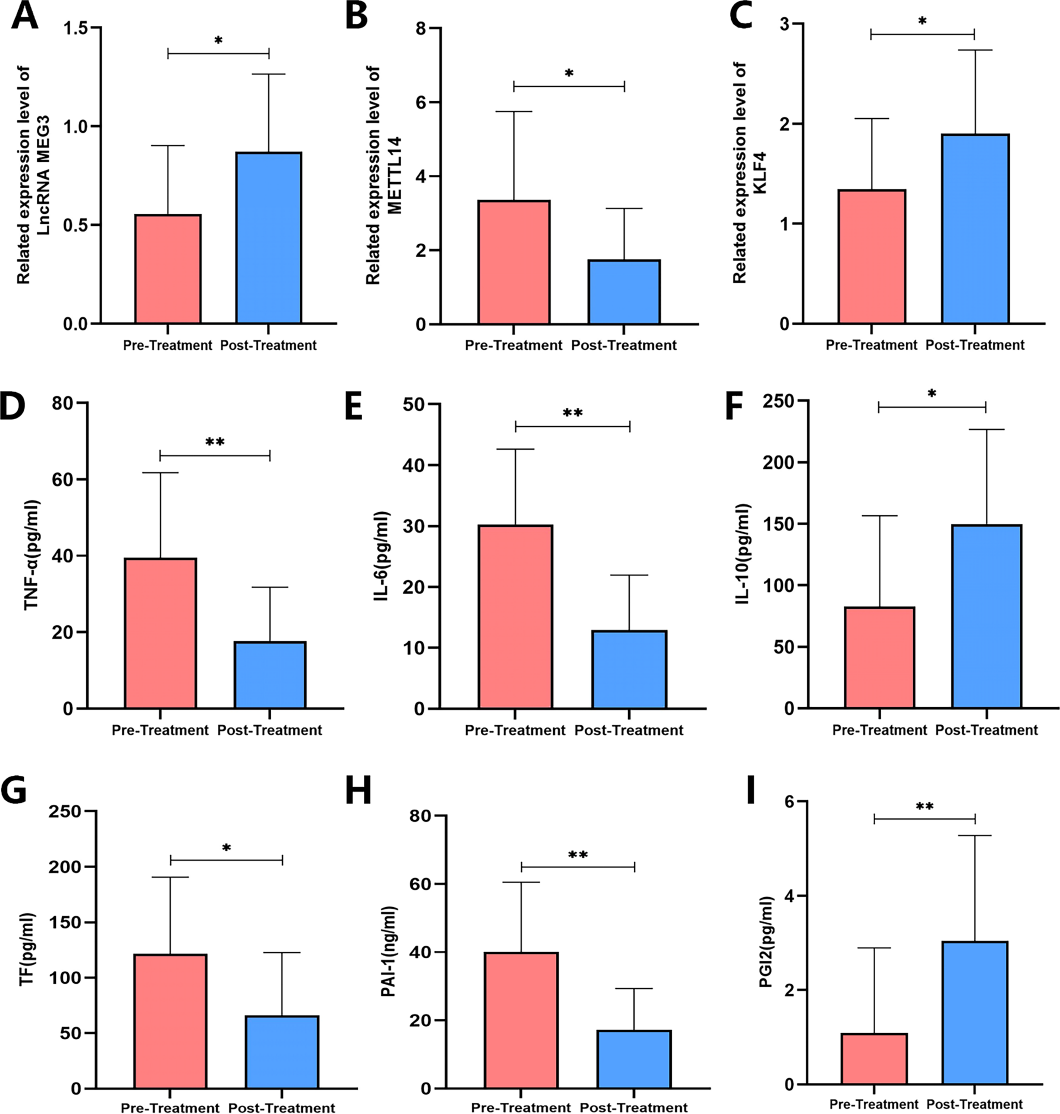
Changes in METTL14, LncRNA MEG3, KLF4, inflammation, and coagulation factors in OA patients before and after treatment. **(A–C)** RT-qPCR method is used to detect the expression of METTL14, LncRNA MEG3, and KLF4; **(D–I)** ELISA detects the levels of TNF - α, IL-6, IL-10, TF, PAI-1, and PGI2. n=30, **P < 0.001,*P < 0.05.

### 3.7 Effects of XFC treatment on METTL14, lncRNA MEG3, KLF4, and inflammatory and coagulation factors in OA patients

To evaluate the effects of XFC treatment on METTL14, lncRNA MEG3, KLF4, as well as inflammatory and coagulation-related factors in patients with OA, PBMCs collected from 30 OA patients after XFC treatment were analyzed and compared with samples obtained before treatment. RT-qPCR analysis showed that, following XFC treatment, the expression levels of lncRNA MEG3 and KLF4 were significantly increased (*p* < 0.05), whereas METTL14 expression was significantly decreased compared with pre-treatment levels (*p* < 0.05) (**Figures 8A–C**). ELISA results further demonstrated that XFC treatment significantly reduced the levels of pro-inflammatory and procoagulant factors, including TNF-α, IL-6, PAI-1, and TF (*p* < 0.05). In contrast, the expression levels of the anti-inflammatory cytokine IL-10 and the anticoagulant factor PGI_2_ were significantly increased after treatment (*p* < 0.05) (**Figures 8D–I**). Collectively, these findings indicate that XFC may regulate the expression of METTL14, lncRNA MEG3, and KLF4 in patients with OA, thereby reducing pro-inflammatory and procoagulant factor levels while enhancing anti-inflammatory and anticoagulant responses.

**Figure 9:**
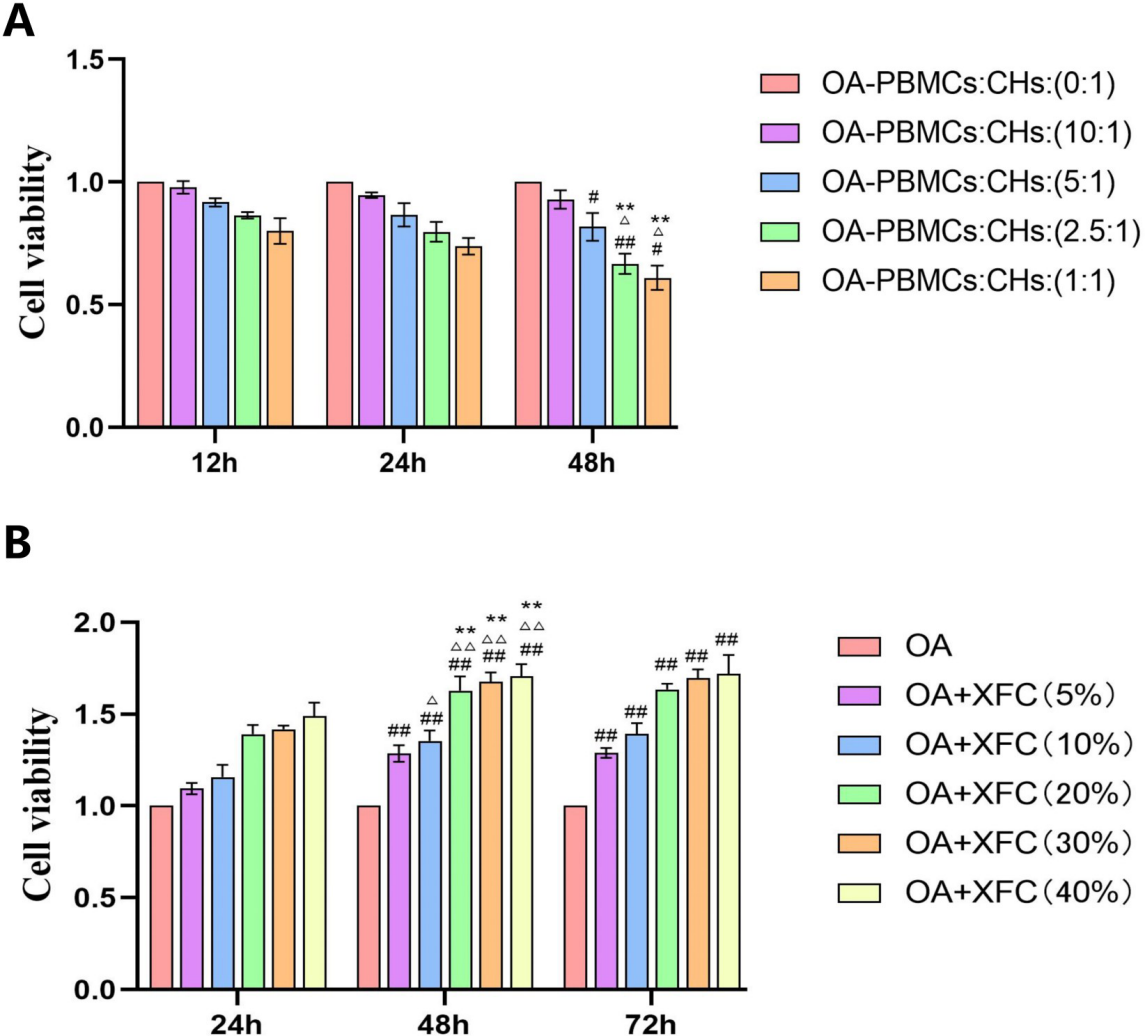
Effect of OA PBMCs and XFC containing serum production of CHs. **(A)** At 48 hours, each proportion group was compared with each proportion group at 12 hours, ^#^p<0.05, ^##^p<0.01; At 48 hours, compare each proportion group with each proportion group at 24 hours, ^△^p<0.05; At 48 hours, the 2.5:1 and 1:1 groups were compared to the 0:1, 10:1, and 5:1 groups, ^**^<0.01. **(B)** Compared with 24h at 48h and 72h for each concentration group, ^##^p<0.01; At 48 hours, each concentration group was compared to the 5% concentration group, ^△^p<0.05, ^△△^p<0.01; At 48 hours, each concentration group was compared to the 10% concentration group, ^**^p<0.01.

### 3.8 Construction of the co-culture model and determination of the optimal concentration and treatment duration of XFC -containing serum

CCK-8 assay was used to evaluate the effects of OA-PBMCs on CH viability at different co-culture ratios (0:1, 10:1, 5:1, 2.5:1, and 1:1) and incubation times (12 h, 24 h, and 48 h). The results showed that, compared with 12 h, there were no significant differences in CH viability among the ratio groups at 24 h (*p*>0.05). At 48 h, CH viability in the 5:1, 2.5:1, and 1:1 ratio groups was significantly decreased (*p* < 0.05). In addition, compared with 24 h, CH viability in the 2.5:1 and 1:1 groups was significantly reduced at 48 h. Therefore, 48h was selected as the optimal co-culture duration. Comparison among the ratio groups at 48 h revealed significant differences in CH viability between the 2.5:1 and 1:1 groups and the 0:1, 10:1, and 5:1 groups (*p* < 0.01), whereas no significant difference was observed between the 2.5:1 and 1:1 groups (*p*>0.05). Consequently, the OA-PBMCs : CHs co-culture model at a ratio of 2.5:1 for 48h was selected for subsequent experiments (**Figure 9A**).

**Figure 10:**
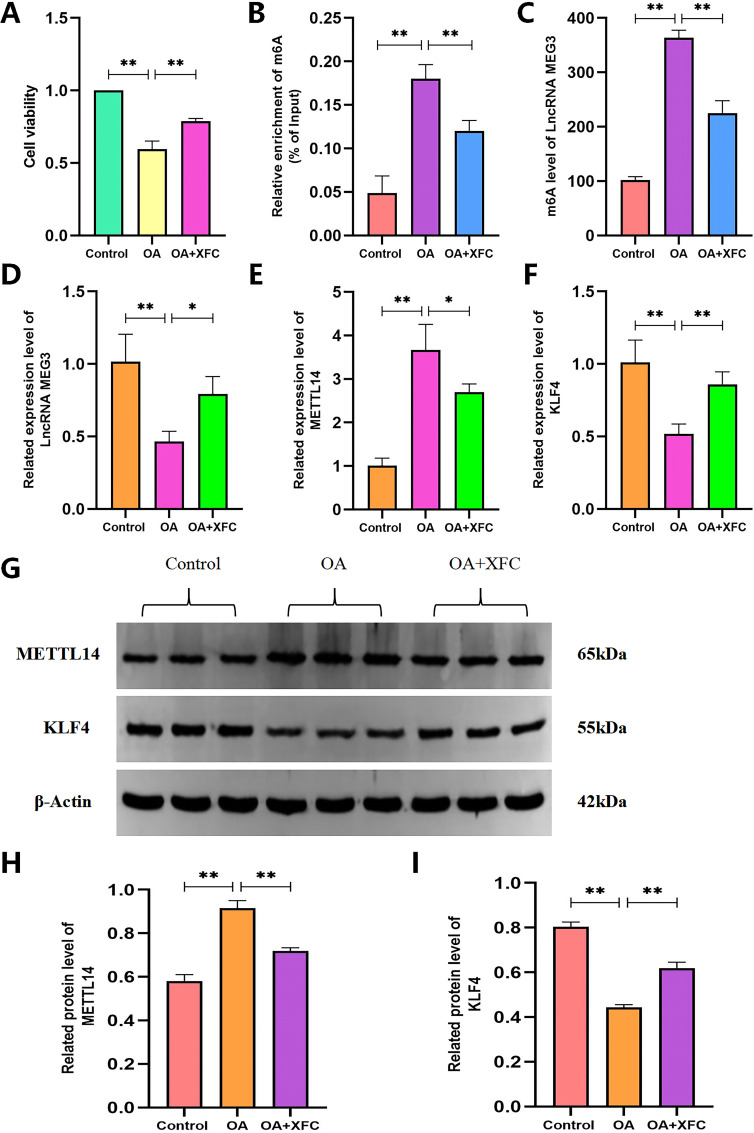
Effects of OA PBMCs and CHs co-culture and XFC on METLLE14/LncRNAMEG3/KLF4. **(A)** CCK8 was used to detect the viability of CHs; **(B)** Colorimetric method was used to detect the total m6A level; **(C)** MeRIP-qPCR was used to detect the level of LncRNA MEG3 m6A; **(D-F)**: RT-qPCR was used to detect the level of LncRNA MEG3, METTL14, KLF4 mRNA levels; **(G–I)** WB was used to detect the level of METTL14 and KLF4 protein expression. n=6, The inter-group comparison was adjusted using Bonferroni multiple comparison,**P < 0.001,*P < 0.05.

The CCK-8 assay was further used to assess the effects of different concentrations (5%, 10%, 20%, 30%, and 40%) and treatment durations (24 h, 48 h, and 72 h) of XFC -containing serum on the viability of co-cultured CHs. The results demonstrated that, compared with 24 h, CH viability at each concentration was significantly increased at 48 h and 72 h (*p* < 0.01). However, no significant differences in CH viability were observed among the different XFC concentration groups at either 48 h or 72 h (*p*>0.05). Therefore, 48 h was selected as the optimal treatment duration. When comparing XFC concentration groups at 48 h, CH viability in the 10%, 20%, 30%, and 40% groups was significantly higher than that in the 5% group (*p* < 0.05). Compared with the 10% group, CH viability in the 20%, 30%, and 40% groups was significantly increased (*p* < 0.05). However, no significant differences were observed among the 20%, 30%, and 40% groups (*p*>0.05). Therefore, a concentration of 20% XFC-containing serum was selected as the optimal concentration for subsequent experiments (**Figure 9B**).

### 3.9 Effects of OA-PBMC-CH co-culture on the METTL14/lncRNA MEG3/KLF4 axis, inflammatory and coagulation factors, and the interventional effects of XFC

**Figure 11:**
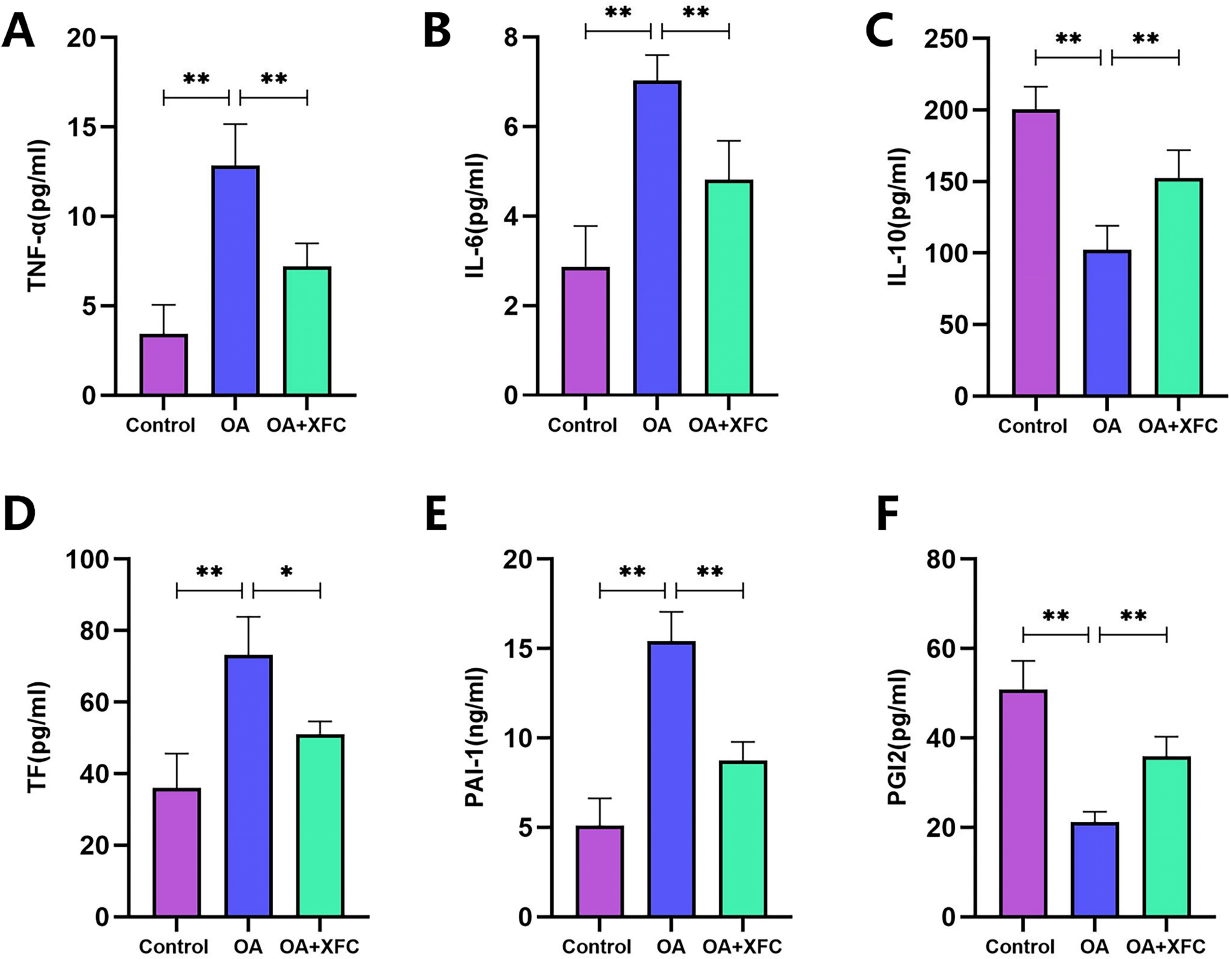
Effects of OA PBMCs and CHs co-culture and XFC on inflammation and coagulation factors. **(A–F)** ELISA was used to evaluate the levels of IL-6, TNF - α, IL-10, PAI-1, TF and PGI2. n=6, The inter-group comparison was adjusted using Bonferroni multiple comparison,**P < 0.001,*P < 0.05.

Using the established co-culture model, we first examined the effects of OA-PBMC stimulation on CH activity. The results demonstrated a significant reduction in CH viability following stimulation with OA- PBMCs (**Figure 10A**). Colorimetric analysis further revealed that CHs stimulated by OA-PBMCsexhibited significantly elevated total m^6^A levels compared with CHs cultured alone (*p* < 0.01) (**Figure 10B**), consistent with the *in vivovivo* findings. MeRIP-qPCR and RT-qPCR analyses showed that the m^6^A modification level of lncRNA MEG3 was significantly increased in the co-culture group (**Figures 10C, D**), while the overall expression level of lncRNA MEG3 was significantly decreased compared with that in CHs alone (*p* < 0.001) (**Figure 10E**). Correspondingly, both mRNA and protein expression levels of METTL14 were significantly elevated in the co-culture group (*p* < 0.001) (**Figures 10E, G, H**), while the mRNA and protein expression levels of KLF4 were markedly reduced (*p* < 0.001) (**Figures 10F, G, I**). ELISA results further showed that stimulation of CHs with OA-PBMCs significantly increased the levels of pro-inflammatory and procoagulant factors, including IL-6, TNF-α, PAI-1, and TF (*p* < 0.001), while the levels of the anti-inflammatory and anticoagulant factors IL-10 and PGI_2_ were significantly decreased (*p* < 0.001) (**Figures 11A-F**). These findings indicate that the co-culture model exhibits pronounced inflammatory and coagulation abnormalities and effectively mimics the *in vivo* pathological environment of OA.

**Figure 12:**
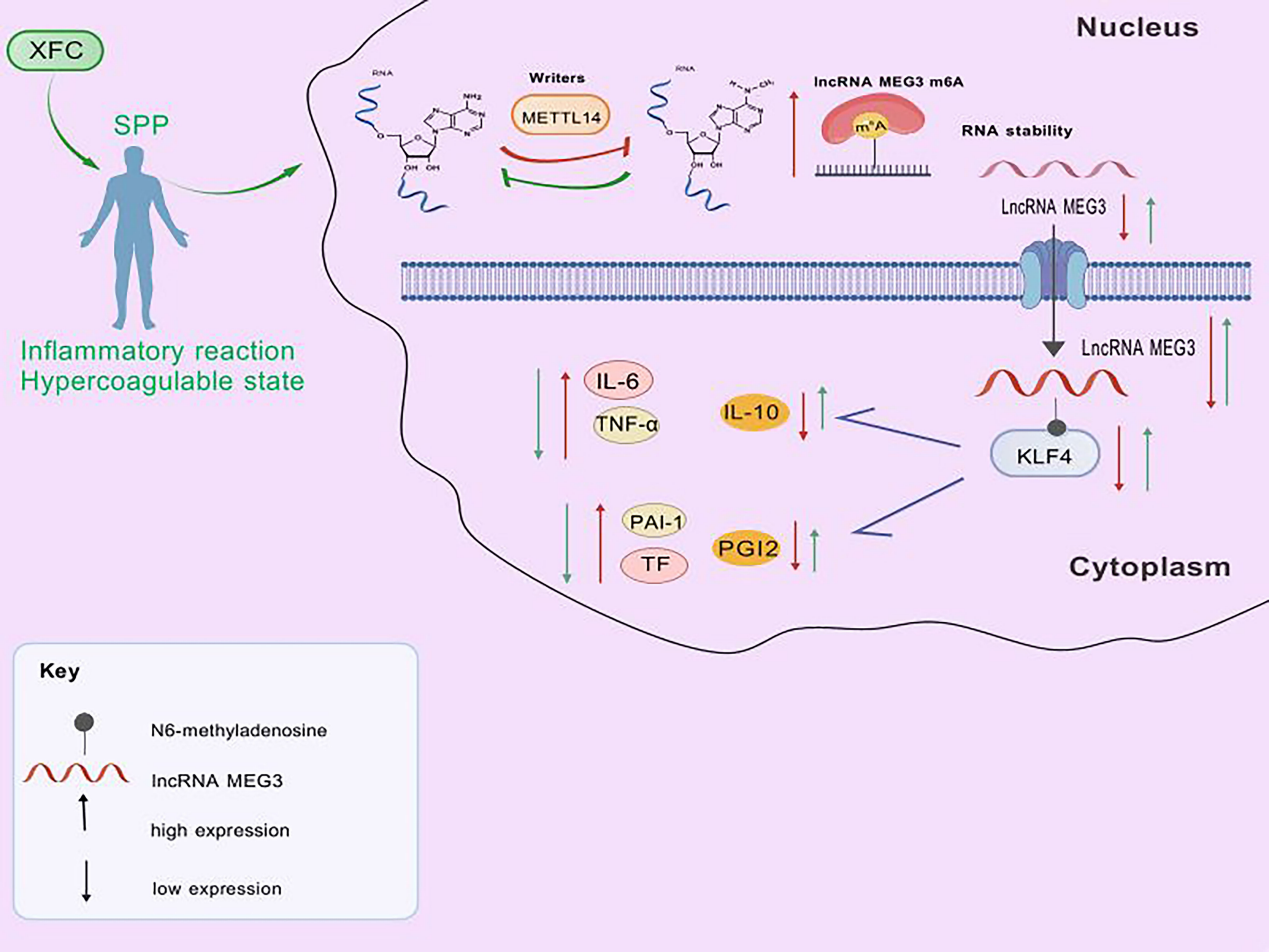
Mechanism diagram of XFC regulating KLF4 through METTL14 mediated LncRNA MEG3 m6A modification to improve hyper-inflammation-associated hypercoagulability and SPP in OA patients.

Subsequently, to evaluate the effects of XFC intervention, XFC-containing serum was added to the OA-PBMC-CH co-culture system. CCK-8 assay results showed that XFC-containing serum significantly reversed the reduction in CH viability induced by OA- PBMC stimulation (**Figure 10A**). Moreover, following XFC intervention, both total m^6^A levels and lncRNA MEG3 m6A modification levels in CHs were significantly reduced (**Figures 10B, C**), while lncRNA MEG3 expression was significantly increased compared with that in the untreated co-culture group (**Figure 10D**). Consistently, the mRNA and protein expression levels of METTL14 were significantly decreased after XFC treatment (*p* < 0.001) (**Figures 10E, G, H**), while the mRNA and protein expression levels of KLF4 were significantly increased (*p* < 0.001) (**Figures 10F, G, I**). ELISA assays further confirmed that XFC -containing serum effectively reversed inflammation- and coagulation-related abnormalities in co-cultured CHs, as evidenced by decreased levels of IL-6, TNF-α, PAI-1, and TF (*p* < 0.001) and increased expression of IL-10 and PGI_2_ (*p* < 0.001) (**Figures 11A-F**). Collectively, these results indicate that XFC may alleviate inflammation and coagulation disorders in OA by regulating METTL14-mediated m^6^A modification of lncRNA MEG3 and subsequently upregulating KLF4 expression.

## 4 Discussion

In this preliminary exploratory study, we comprehensively integrated analyses of public databases, clinical patient data, and *in vitro* experiments to confirm the presence of hyperinflammation-associated hypercoagulability and abnormalities in SPP in individuals with OA. Our findings suggest that XFC may improve hyperinflammation-associated hypercoagulability and SPP in OA patients by regulating KLF4 expression through METTL14-mediated m^6^A modification of lncRNA MEG3 (**Figure 12**).

### 4.1 Hyperinflammation-associated hypercoagulability represents a critical link in the initiation and progression of OA

A hypercoagulable state, also referred to as hypercoagulability or a pre-thrombotic state, is characterized by excessive activation of coagulation factors, leading to abnormal blood clot formation (37). Previous studies have demonstrated that pro-inflammatory cytokines, such as TNF- α and IL-6, promote extracellular matrix degradation in CHs, resulting in joint tissue damage and concomitant injury to vascular endothelial cells. These inflammatory mediators can directly or indirectly activate the coagulation-fibrinolysis system and disrupt anticoagulant mechanisms, leading to microcirculatory dysfunction, systemic circulatory abnormalities, and ultimately the development of a hypercoagulable state in patients with OA (6, 7, 38). Vascular endothelial cells (VECs) line the inner surface of all blood vessels and serve as a crucial barrier for circulating blood (39, 40). Under physiological conditions, VECs inhibit the activation of coagulation factors, promote fibrinolytic activity, prevent thrombosis, and maintain vascular patency (41, 42). However, when VECs are damaged by various stimuli, including inflammatory factors, their structure and function are impaired. This damage triggers the release of antifibrinolytic substances, such as PAI-1 and TF, while suppressing the release of anticoagulant factors, including tissue-type plasminogen activator (tPA). Consequently, anticoagulant properties on the endothelial surface are lost, and VECs undergo a phenotypic shift from an anticoagulant to a procoagulant state, thereby promoting thrombus formation (43). In addition, inflammatory mediators can induce the release of vasoactive substances from VECs, activate platelets, increase circulating procoagulant factors, inhibit anticoagulant factor release, and reduce fibrinolytic system activity. Together, these processes enhance blood coagulation and contribute to systemic hypercoagulability (44).

Laboratory examinations are essential for diagnosing a hypercoagulable state, and commonly used indicators include FBG, PLT, APTT, TT, and PT. Abnormal elevations in these parameters indicate a hypercoagulable state or an increased tendency toward thrombosis (45). In the present study, analysis of laboratory indices and PBMCs from 30 patients with OA revealed that inflammatory markers, including ESR and hs-CRP, were significantly elevated. Coagulation-related indicators, such as PLT, FBG, and D-dimer, were also markedly increased. In addition, the expression levels of pro-inflammatory cytokines TNF- α and IL- 6 in PBMCs from OA patients were significantly increased, whereas the expression level of the anti-inflammatory cytokine IL- 10 was significantly reduced (*p* < 0.01). Furthermore, the expression levels of procoagulant factors PAI-1 and TF were significantly elevated (*p* < 0.01), while the level of the anticoagulant factor PGI_2_ was significantly decreased (*p* < 0.01).

### 4.2 LncRNA MEG3 and KLF4 are likely to be involved in the regulation of hyperinflammation-associated hypercoagulability in OA

The role of lncRNA MEG3 in modulating the inflammatory hypercoagulable state in OA has been preliminarily demonstrated (16). Previous studies have shown that lncRNA MEG3 is downregulated in patients with OA and promotes the release of pro-inflammatory and procoagulant cytokines by activating the NF-κB signaling pathway, while inhibiting the production of anti-inflammatory and anticoagulant cytokines. In addition, reduced lncRNA MEG3 expression suppresses CH viability and exacerbates inflammatory responses. In the present study, analysis of PBMCs from the enrolled population further confirmed that lncRNA MEG3 is significantly downregulated in OA patients and exhibits potential as a molecular biomarker to aid in OA diagnosis. Moreover, our findings demonstrate that KLF4, as a downstream target of lncRNA MEG3, plays a crucial role in regulating inflammatory responses and the initiation and progression of the hypercoagulable state in OA.

KLF4 is a zinc finger transcription factor that is widely involved in the regulation of vascular endothelial cell functions, including inflammatory responses, angiogenesis, coagulation, and maintenance of vascular integrity (46). Previous studies have demonstrated that KLF4 can regulate the expression of the inflammatory mediator IL-6 and indirectly influence coagulation function by suppressing inflammatory responses (47). In addition, KLF4 protects endothelial function and ameliorates hypercoagulability by inhibiting NF-κB activity, thereby reducing inflammatory cell adhesion and the secretion of pro-inflammatory mediators (48). A Furthermore, a study based on the GEO database that identified and validated aging-related genes in OA reported that KLF4 is significantly downregulated in OA, suggesting that it may serve as a novel biomarker associated with OA aging and a potential therapeutic target for OA treatment (49). These findings are consistent with the results of the present study.

### 4.3 METTL14 mediated lncRNA MEG3 m^6^A modification is of significant importance in hyperinflammation-associated hypercoagulability in OA

m^6^A modification refers to the methylation of the nitrogen atom at the sixth position of adenosine (A) in RNA molecules (50, 51). As the most prevalent form of post-transcriptional modification, m^6^A modification occurs throughout the RNA life cycle, including splicing, processing, translation, and degradation, and is extensively involved in physiological and pathological processes, closely related to the initiation and progression of various diseases (52). M^6^ A modification is a dynamic and reversible process that is primarily regulated by the coordinated actions of three classes of proteins: writers (methyltransferases), erasers (demethylases), and readers (m^6^A-binding proteins). In the nucleus, methyltransferases catalyze the methylation of the sixth nitrogen atom of adenosine on RNA, whereas demethylases remove m^6^A marks, thereby influencing mRNA splicing and other nuclear events. After export to the cytoplasm, reader proteins recognize m^6^A-modified RNA and regulate downstream biological processes, including RNA degradation, translation, localization, and cleavage, ultimately affecting RNA stability and translational efficiency (12). METTL14 is a core m^6^A methyltransferase that plays a critical role in eukaryotic cells by forming a heterodimeric complex with METTL3 to catalyze m^6^A deposition (53, 54). Previous studies have reported that METTL14 expression is significantly increased in OA cell models induced by IL-1β stimulation of human CHs, as well as in OA animal models established by medial collateral ligament and medial meniscus transection. Furthermore, knockout of METTL14 has been shown to inhibit extracellular matrix degradation and CH ferroptosis in an m^6^A-dependent manner (55). Consistent with these findings, our results demonstrate that METTL14 expression is significantly upregulated in PBMCs from patients with OA and in OA-PBMC-CH co-culture models, and that METTL14 expression is strongly correlated with the downregulation of lncRNA MEG3.

### 4.4 The regulatory effect of XFC on inflammatory response, hypercoagulability, and SPP in OA patients

SPP refers to an individual’s self- evaluation of adaptation to physical, psychological, and social conditions, reflecting the gap between personal expectations and actual living circumstances (30). A larger gap indicates poorer quality of life and a less favorable patient experience. The self-assessment scale for patients with OA used in this study integrates the SF-36 Health Survey, VAS, SAS, SDS, and a TCM symptom scoring system. This scale provides quantitative assessments of disease activity, joint function, anxiety and depression, and TCM-related symptoms. By combining multidimensional evaluation methods from both Western and TCM, the scale can more objectively reflect changes in patients’ conditions. Comparison of scores before and after treatment enables accurate assessment of therapeutic efficacy and improves the precision of clinical syndrome differentiation and treatment strategies. The effectiveness of this scale has been validated in multiple previous clinical studies (29). Analysis of the scale data revealed that OA patients exhibited marked deterioration in SPP, characterized by significant reductions across all eight dimensions of the SF-36 and significant increases in SAS, SDS, and VAS. Notably, TCM syndrome assessment showed that scores for CDS, SDH, SBS, and SDSSD were elevated to varying degrees, indicating abnormal SPP in patients with active OA and substantial impairment of quality of life. After treatment, SPP scores in OA patients were significantly improved. Association rule analysis further demonstrated that XFC was significantly associated with reductions in SAS, SDS, SDSSD, and SBS scores and showed a strong correlation with increased RE scores (support>50%, confidence>70%, lift>1). The association rule network revealed a strong relationship between XFC treatment and improvements in SPP scores, as well as reductions in inflammatory and coagulation-related indicators in OA patients. These findings confirm that XFC exerts significant therapeutic effects in alleviating inflammatory responses, improving hypercoagulable states, and enhancing SPP in patients with OA. Furthermore, analyses of PBMCs and co-cultured CHs before and after treatment demonstrated that XFC intervention significantly reduced METTL14 expression and the levels of pro-inflammatory and procoagulant factors, while increasing the expression of lncRNA MEG3, KLF4, and anti-inflammatory and anticoagulant factors in OA.

### 4.5 Research advantages, limitations, and future outlook

Several important strengths of this study merit consideration. First, XFC has a well-established pharmacological and clinical research foundation, which underscores the rigor of its formulation and supports its clinical effectiveness. Second, while most existing studies investigating the role of m6A modification in OA rely primarily on bioinformatics analyses and sequencing data, they often lack validation using clinical patient specimens. By integrating clinical data with experimental validation, the present study not only elucidates a potential mechanism by which XFC may regulate KLF4 expression through METTL14-mediated m6A modification of lncRNA MEG3 to improve inflammation and hypercoagulability in OA patients, but also verifies its clinical feasibility. These findings provide a viable TCM-based diagnostic and therapeutic strategy for patients with OA-associated hypercoagulability.

However, several limitations of this study should be acknowledged. First, all clinical samples were obtained from a single center, the First Affiliated Hospital of Anhui University of Traditional Chinese Medicine, and the sample size for both clinical and experimental analyses was relatively small. This limited sample size may reduce the statistical power of the study and increase the risk of overinterpretation of the results. In future research, we plan to expand the sources of clinical samples, collect data from a larger patient population, and validate the generalizability of our findings through independent, large- scale cohort studies. These efforts will further clarify the clinical efficacy of XFC and strengthen the evidence supporting its use in the treatment of OA. In addition, although several potential confounding factors were controlled through rigorous study design, the enrolled patients were relatively elderly and commonly presented with multiple comorbidities and histories of concomitant medication use. These factors may act as residual confounders and influence the study outcomes. Therefore, future studies should perform stratified analyses based on larger sample sizes to further minimize the impact of confounding variables.

Finally, as a multi-component compound preparation, the therapeutic efficacy of XFC is likely attributable to the synergistic regulatory network of its multiple constituents. However, the present study did not identify the specific active components responsible for modulating METTL14 or m^6^A modification. In future work, analytical approaches such as liquid chromatography-mass spectrometry (LC- MS) and cellular thermal shift assays will be employed to identify the key bioactive components of XFC that target METTL14 or m^6^A-related pathways.

## 5 Conclusions

In this study, we integrated public database analyses, clinical peripheral blood assessments from patients, and *in vitro* experimental validation to explore the potential involvement of METTL14-mediated m^6^A modification of lncRNA MEG3 in the occurrence and progression of hyperinflammation-associated hypercoagulability in OA from the perspective of lncRNA m^6^A regulation. As a potential therapeutic strategy, XFC may exert beneficial effects on hyperinflammation-associated hypercoagulability in OA by modulating METTL14/lncRNA MEG3 m^6^A modification. As a preliminary exploratory investigation, this study verifies the potential role of the METTL14–lncRNA MEG3–KLF4 regulatory axis in OA and the interventional effects of XFC from both clinical and *in vitro* experimental perspectives through correlation analyses. These findings generate promising hypotheses and provide preliminary yet robust evidence and directional support for subsequent *in vivo* animal studies and direct functional validation. In future work, rescue experiments involving knockdown or overexpression of METTL14 will be conducted to further elucidate the regulatory relationships between METTL14 and downstream cytokines, thereby validating and extending the conclusions of the present study.

## Data Availability

The datasets presented in this study can be found in online repositories. The names of the repository/repositories and accession number(s) can be found in the article/**Supplementary Material**.
