# Supplementary material for: Xinfeng capsule improves hyperinflammation-associated hypercoagulability and self-perception in osteoarthritis by regulating KLF4 through METTL14-mediated m6A modification of lncRNA MEG3
Source: Front Immunol. 2026 Jan 29;17:1749727. doi: 10.3389/fimmu.2026.1749727 (PMC12894041; doi:10.3389/fimmu.2026.1749727)
Supplement: Supplementary file 1 [file DataSheet1.pdf]

国 家 中 医 药 高 水 平 重 点 学 科

—中医痹病学科

国家临床重点专科中医风湿病科

骨关节炎患者感受

自评量表

患者姓名：______________

住 院 号：______________

填 表 人：______________

填表时间：______________

风 湿 科 患 者 自 评 量 表

姓名： 科别： 病区： 床号： 住院号：

尊敬的患者您好，本量表是专门设计用来评估每位风湿病患者的综合情况。本问卷请由患者本人或患者家属认真、如实的填写。请认真阅读以下各个项目，将其中最符合您情况的答案勾出。如有无法理解的问题，请咨询医务人员，在指导下进行正确填写，这将对医生了解病情和指导康复治疗有很大的帮助。您的资料和标本用于病情评估，还可能用于科学研究。谢谢您的参与配合！

患者一般情况：

姓名：	性别：	年龄： 岁	测定日期： 年 月 日	第 次测定
身高： CM	体重： KG	病程： 年	诊断：	

是否长期吸烟：是/否 烟龄：

是否长期饮酒：是/否 酒龄：

是否患有其他疾病：是/否 疾病名称：

是否曾就诊于其他医院：是/否 其他医院名：

是否曾服用其他药物：是/否 其他药物名：

其他：

第一部分 健康调查简表SF-36

SF-36, 健康调查简表(the MOS item short from health survey, SF-36)，是在1988年 Stewartse研制的医疗结局研究量表(medical outcomes study –short from, MOS SF)的基础上，由美国波士顿健康研究发展而来。1991年浙江大学医学院社会医学教研室翻译了中文版的SF-36。

SF-36量表的内容：

1、总体来讲，您的健康状况是： 条目得分：

①非常好 ②很好 ③好 ④一般 ⑤差

2、跟1年以前比您觉得自己的健康状况是： 条目得分：

①比1年前好多了 ②比1年前好一些 ③跟1年前差不多 ④比1年前差一些 ⑤比1年前差多了

健康和日常活动

3、以下这些问题都和日常活动有关。请您想一想，您的健康状况是否限制了这些活动？如果有限制，程度如何？

（1）重体力活动。如跑步举重、参加剧烈运动等： 条目得分：

①限制很大 ②有些限制 ③毫无限制

（2）适度的活动。如移动一张桌子、扫地、打太极拳、做简单体操等： 条目得分：

①限制很大 ②有些限制 ③毫无限制

（3）手提日用品。如买菜、购物等： 条目得分：

①限制很大　②有些限制　③毫无限制

（4）上几层楼梯： 条目得分：

①限制很大　②有些限制　③毫无限制

（5）上一层楼梯： 条目得分：

①限制很大　②有些限制　③毫无限制

（6）弯腰、屈膝、下蹲： 条目得分：

①限制很大　②有些限制　③毫无限制

（7）步行1500米以上的路程： 条目得分：

①限制很大　②有些限制　③毫无限制

（8）步行1000米的路程： 条目得分：

①限制很大　②有些限制　③毫无限制

（9）步行100米的路程： 条目得分：

①限制很大　②有些限制　③毫无限制

（10）自己洗澡、穿衣： 条目得分：

①限制很大　②有些限制　③毫无限制

4、在过去4个星期里，您的工作和日常活动有无因为身体健康的原因而出现以下这些问题？

（1）减少了工作或其他活动时间： 条目得分：

①是　②不是

（2）本来想要做的事情只能完成一部分： 条目得分：

①是　②不是

（3）想要干的工作或活动种类受到限制： 条目得分：

①是　②不是

（4）完成工作或其他活动困难增多（比如需要额外的努力）: 条目得分：

①是　②不是

5、在过去4个星期里，您的工作和日常活动有无因为情绪的原因（如压抑或忧虑）而出现以下这些问题？

（1）减少了工作或活动时间： 条目得分：

①是　②不是

（2）本来想要做的事情只能完成一部分： 条目得分：

①是　②不是

（3）干事情不如平时仔细： 条目得分：

①是　②不是

6、在过去4个星期里，您的健康或情绪不好在多大程度上影响了您与家人、朋友、邻居或集体的正常社会交往？ 条目得分：

①完全没有影响　②有一点影响　③中等影响　④影响很大　⑤影响非常大

7、在过去4个星期里，您有身体疼痛吗？ 条目得分：

①完全没有疼痛　②有很轻微疼痛　③有轻微疼痛　④有中度疼痛　⑤有严重疼痛　⑥有很严重疼痛

8、在过去4个星期里，您的身体疼痛影响了您的工作和家务吗？　条目得分：

①完全没有影响　②有一点影响　③中等影响　④影响很大　⑤影响非常大

9、以下这些问题是关于过去1个月里您自己的感觉，对每一条问题所说的事情，您的情况是什么样的？

（1）您觉得生活充实：　条目得分：

①所有的时间　②大部分时间　③比较多时间　④一部分时间　⑤小部分时间　⑥没有这种感觉

（2）您是一个敏感的人：　条目得分：

①所有的时间　②大部分时间　③比较多时间　④一部分时间　⑤小部分时间　⑥没有这种感觉

（3）您的情绪非常不好，什么事都不能使您高兴起来：　条目得分：

①所有的时间　②大部分时间　③比较多时间　④一部分时间　⑤小部分时间　⑥没有这种感觉

（4）您的心理很平静：　条目得分：

①所有的时间　②大部分时间　③比较多时间　④一部分时间　⑤小部分时间　⑥没有这种感觉

（5）您做事精力充沛：　条目得分：

①所有的时间　②大部分时间　③比较多时间　④一部分时间　⑤小部分时间　⑥没有这种感觉

（6）您的情绪低落：　条目得分：

①所有的时间　②大部分时间　③比较多时间　④一部分时间　⑤小部分时间　⑥没有这种感觉

（7）您觉得筋疲力尽：　条目得分：

①所有的时间　②大部分时间　③比较多时间　④一部分时间　⑤小部分时间　⑥没有这种感觉

（8）您是个快乐的人：　条目得分：

①所有的时间　②大部分时间　③比较多时间　④一部分时间　⑤小部分时间　⑥没有这种感觉

（9）您感觉厌烦：　条目得分：

①所有的时间　②大部分时间　③比较多时间　④一部分时间　⑤小部分时间　⑥没有这种感觉

10、不健康影响了您的社会活动（如走亲访友）：　条目得分：

①所有的时间　②大部分时间　③比较多时间　④一部分时间　⑤小部分时间　⑥没有这种感觉

总体健康情况

11、请看下列每一条问题，哪一种答案最符合您的情况？

（1）我好象比别人容易生病：　条目得分：

①绝对正确　②大部分正确　③不能肯定　④大部分错误　⑤绝对错误

（2）我跟周围人一样健康：　条目得分：

①绝对正确　②大部分正确　③不能肯定　④大部分错误　⑤绝对错误

（3）我认为我的健康状况在变坏：　条目得分：

①绝对正确　②大部分正确　③不能肯定　④大部分错误　⑤绝对错误

（4）我的健康状况非常好：　条目得分：

①绝对正确　②大部分正确　③不能肯定　④大部分错误　⑤绝对错误

生理机能得分：	生理职能得分：	躯体疼痛得分：
一般健康状况得分：	精力得分：	社会功能得分：
情感职能得分：	精神健康得分：	健康变化得分：

第二部分 关节疼痛症状评价调查表(VAS)

1、关节疼痛：采用VAS（Visual Analog Scales）评分。

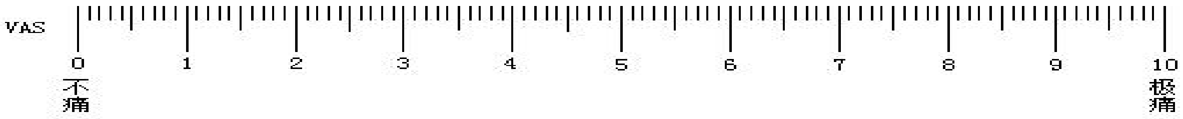

关节疼痛VAS评分：

2、患者对疾病活动的整体评估：采用VAS（Visual Analog Scales）评分

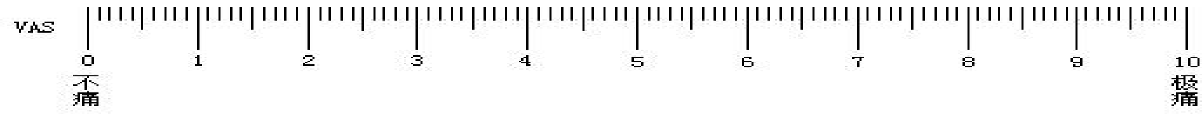

患者对疾病活动的整体评估VAS评分：

3、医生对疾病活动的整体评估：采用VAS（Visual Analog Scales）评分

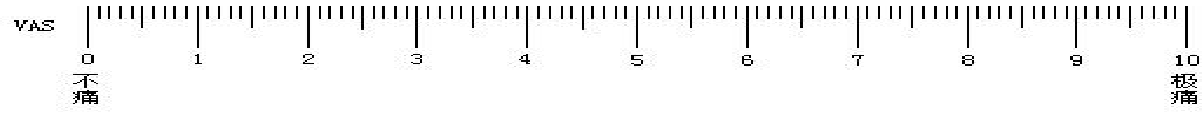

医生对疾病活动的整体评估评分：

4、请在图中大致圈出您所患关节疼痛的位置

左侧关节疼痛位置：

右侧关节疼痛位置：

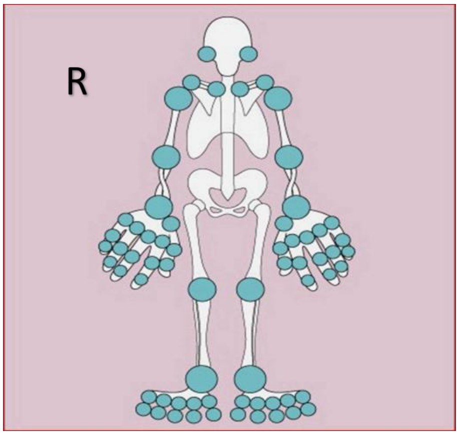

风 湿 科 患 者 自 评 量 表

姓名：　　　　科别：　　　　病区：　　　　床号：　　　　住院号：

第三部分 焦虑自评量表（SAS）评价

指导语：下面有二十条文字，请您仔细阅读每条项目，明确了解各项问题的意思，然后根据你最近一周的实际感觉，进行选择：（由患者本人选择填写）

评定项目	没有或很少有	有时有	大部分时间有	绝大多数时间有	评分
1.我感到比往常更加精神过敏和焦虑	①	②	③	④	
2.无缘无故感到担心	①	②	③	④	
3.容易心烦意乱或感到恐慌	①	②	③	④	
4.感到我的身体好像被分成几块，支离破碎	①	②	③	④	
5.感到事事都很顺利,不会有倒霉的事情发生	①	②	③	④	
6.四肢抖动和震颤	①	②	③	④	
7.因头痛、颈痛和背痛而烦恼	①	②	③	④	
8.感到无力而且容易疲劳	①	②	③	④	
9.感到很平静,能安静下来	①	②	③	④	
10.我感到我的心跳较快	①	②	③	④	
11.我因阵阵的眩晕而不舒服	①	②	③	④	
12.有阵阵要昏倒的感觉	①	②	③	④	

13.呼吸时进气和出气都不费力	①	②	③	④	
14.手指和脚趾感到麻木和刺痛	①	②	③	④	
15.因胃痛和消化不良而苦恼	①	②	③	④	
16.必须时常排尿	①	②	③	④	
17.我的手总是温暖而干燥	①	②	③	④	
18.觉得脸发烧发红	①	②	③	④	
19.容易入睡,晚上休息很好	①	②	③	④	
20.做恶梦	①	②	③	④	

评测结果:

评分方法：

SAS标准分的分界值为50分，其中50—59分为轻度焦虑，60—69分为中度焦虑，70分以上为重度焦虑。

SAS采用4级评分，主要评定症状出现的频度，其标准为：“1”表示没有或很少时间有；“2”表示有时有；“3”表示大部分时间有；“4”表示绝大部分或全部时间都有。20个条目中有l5项是用负性词陈述的，按上述l～4顺序评分。其余5项（第5，9，13，17，19）注*号者，是用正性词陈述的，按4～1顺序反向计分。

把各题的得分相加为总分，总分乘以1.25，四舍五入取整数即得到标准分。

风 湿 科 患 者 自 评 量 表

姓名：　　　　　科别：　　　　　病区：　　　　　床号：　　　　　住院号：

第四部分 抑郁自评量表（SDS）

指导语：下面有二十条文字，请您仔细阅读每条项目，明确了解各项问题的意思，然后根据你最近一周的实际感觉，进行选择：（由患者本人选择填写）

评定项目	没有或很少有	有时有	大部分时间有	绝大多数时间有	评分
1.我觉得闷闷不乐,情绪低沉	①	②	③	④	
2.觉得一天之中早晨最好	①	②	③	④	
3.一阵阵地哭出来或者觉得想哭	①	②	③	④	
4.晚上睡眠不好	①	②	③	④	
5.吃的跟平常一样多	①	②	③	④	
6.与异性密切接触时和以往一样感到愉快	①	②	③	④	
7.发觉我的体重下降	①	②	③	④	
8.有便秘的苦恼	①	②	③	④	
9.心跳比平时快	①	②	③	④	
10.无缘无故感到疲乏	①	②	③	④	
11.头脑跟平常一样清楚	①	②	③	④	
12.觉得做以前经常做的事并没有困难	①	②	③	④	
13.觉得不安而平静不下来	①	②	③	④	

14. 对将来抱有希望	①	②	③	④	
15. 比平常容易激动	①	②	③	④	
16. 觉得做出决定是容易的	①	②	③	④	
17. 觉得自己是个有用的人,有人需要我	①	②	③	④	
18. 我的生活过得很有意思	①	②	③	④	
19. 认为如果我死了别人会生活得好些	①	②	③	④	
20. 平常感兴趣的事我仍然照样感兴趣	①	②	③	④	

评测结果:

评分方法：

SDS 的总分小于50分为无抑郁；大于等于50分且小于60分为轻微至轻度抑郁；大于等于60分且小于70分为中至重度抑郁；标准分大于等于70分为重度抑郁。

SDS采用4级评分，评分时间为过去一周内。主要评定症状出现的频度，其标准为："1"表示没有或很少时间有；"2"表示有时有；"3"表示大部分时间有；"4"表示绝大部分或全部时间都有。

SDS 的总分等于各条目得分之和，其中第2、5、6、11、12、14、16、17、18和20题为反序记分，按4～1顺序反向计分。其余诸向按上述l～4顺序评分。

把各题的得分相加为总分，总分乘以1.25，四舍五入取整数即得到标准分。

第五部分　中医证候量化积分

1、“寒痹”

主症：肢体关节冷痛，肿胀或重着，局部皮色不红，触之不热，晨僵，关节屈伸不利，遇寒痛剧，得温痛减，局部畏寒怕风，或恶风发热，肌肤麻木不仁。

次症：或口淡不渴，恶风寒，阴雨天加重，肢体沉重，头身困重，舌质淡或淡红，苔薄白或白腻，脉弦紧或沉紧或浮缓

主症					
症 状	0 分	2 分	4 分	6 分	积分
关节冷痛	无明显关节冷痛	关节作冷仅关节恶风寒，触之不凉	关节恶风寒，触之凉，喜温	关节恶风寒明显，常加衣保护	
关节肿胀	不肿胀	轻度肿胀，皮肤纹理变浅，关节的骨标志仍明显	关节肿胀，皮肤纹理基本消失，骨标志不明显	关节肿胀甚，皮肤紧，骨标志消失	
关节重着	无	轻度关节重着，屈伸不利，活动后缓解	关节重着，活动不利，活动后缓解不明显	关节重着僵硬，活动受限，活动后不能缓解	
晨僵	无	少于 1 小时	1 小时至 2 小时	大于 2 小时	
次症					
症状	0 分	1 分	2 分	3 分	积分
畏恶风寒	无	偶有畏恶风寒	经常畏恶风寒，不需加衣	常畏恶风寒，需加衣	
肢冷	无	肢冷不温自觉时冷，但触之不凉	经常自觉冷，触之凉	肢冷重伴有疼痛	
怯寒	无	微恶风	怯寒不加衣	怯寒要加衣	
头身困重	无	稍觉困重，不影响活动	困重较明显，活动减少	困重明显，不微活动	
口淡不渴	无	偶觉口淡乏味	时觉口淡无味，饮水较平日减少	持续口谈无味，整日无口渴	

2、“热痹”

主症:四肢关节或肌肉局部红肿，重着，疼痛如燎，局部肤温升高，下肢关节尤甚，晨僵，活动受限，或关节积液，屈伸不利，或伴发热、心烦口渴。

次症：口苦口粘，口渴不欲饮，恶心，或恶风发热，有汗不解，或肢体困重身热不扬，便干溲黄，舌红，苔黄腻或燥，脉滑数或弦滑。

主症					
**症状**	**0 分**	**2 分**	**4 分**	**6 分**	**积分**
**关节热痛**	无明显自觉发热	关节发热仅关节触之热	关节触之热，伴有自觉热	关节触之热，伴自觉灼热	
**关节重着**	无	轻度关节重着，屈伸不利，活动后缓解	关节重着，活动不利，活动后缓解不明显	关节重着僵硬，活动受限，活动后不能缓解	
**晨僵**	无	少于 1 小时	1 小时至 2 小时	大于 2 小时	
**发热**	37℃以下	37.5-37.9℃	38-38.9℃	39℃以上	
**口渴**	无	偶有口渴	经常口渴，常需饮水	口渴频饮	
**次症**					
**症状**	**0 分**	**1 分**	**2 分**	**3 分**	**积分**
**汗出**	无	偶有汗出多	经常汗多，动则汗出	汗出较多，常湿衣襟	
**口腻**	无	偶觉口腻	时觉口中粘腻	持续口中粘腻	
**恶心**	无	偶有恶心	时有恶心，偶有欲呕	频频恶心，有时欲呕甚或呕	
**肢体困重身热不扬**	无	稍觉困重，不影响活动	困重较明显，活动减少觉发热，按之稍热	全身发热，按之较热	

3、“脾虚湿盛”

主症					
**症 状**	**0 分**	**2 分**	**4 分**	**6 分**	**积分**
**关节重着**	无	轻度关节重着，屈伸不利，活动后缓解	关节重着，活动不利，活动后缓解不明显	关节重着僵硬，活动受限，活动后不能缓解	
**皮下硬结**	无	轻度硬节，风湿结节在 2 个以内	中度硬节，2~4 个风湿结节	重度硬节，4 个以上风湿结节	
**食少纳呆**	无	没有食欲，但保持原饭量	无食欲，饭量比病前减少 1/3	饭量减少 2/3 以上	
**少气懒言**	无	精神不振，不喜多言，不问不答	精神疲乏，思睡，懒于言语	精神极度疲乏，偶语	
**食后腹胀**	无	轻微腹胀，半小时后减轻或消失	腹胀不适持续 1 个小时，影响日常生活，或需对症处理	腹胀不适持续 2 个小时以上，服药处理效果不好	
**次症**					
**症状**	**0 分**	**1 分**	**2 分**	**3 分**	**积分**
**倦怠乏力**	无	稍倦不耐劳力，可坚持轻体力劳动	倦怠较甚，勉强支持日常活动	四肢无力，不能坚持日常活动	
**大便稀溏**	大便正常	软便或稍烂，成堆不成形，1~2 次/天	烂便，溏便 4~5 次/天，或稀便 1~2 次/天	稀便，每日 3 次以上	
**头身困重**	无	稍觉困重不影响活动	困重较明显，活动减少	困重明显，不欲活动	

第六部分　中医血瘀证量化积分

姓名：　　　　　科别：　　　　　病区：　　　　　床号：　　　　　住院号：

主要症状			
症状	2 分	症状	2 分
舌质紫暗<br>瘀斑瘀点		间歇性跛行	
面部、口唇、眼周、指（趾）端青紫暗黑		腹部压痛抵抗感	
静脉曲张或<br>毛细血管异常扩张		闭经或月经暗黑有块	
出血后引起脏器、组织、皮下瘀血或积血		血管闭塞或<br>中度狭窄＞50%<br>血栓、梗塞或栓塞	
次要症状			
症状	1 分	症状	1 分
固定性疼痛<br>或刺痛、绞痛<br>或入夜尤甚		脉涩或结代或无脉	
肢体麻木或偏瘫		脏器肿大、新生物、炎性或非炎性包块、组织增生	
痛经		影像学检查显示<br>血管狭窄＜50%	
肌肤甲错		血液流变性、凝血、纤溶、微循环等检测异常	
精神狂躁或善忘		近 1 个月有<br>外伤、手术或人工流产	

注：符合主要症状1条或次要症状2条即可诊断血瘀证
