# Supplementary material for: Xinfeng capsule improves hyperinflammation-associated hypercoagulability and self-perception in osteoarthritis by regulating KLF4 through METTL14-mediated m6A modification of lncRNA MEG3
Source: Front Immunol. 2026 Jan 29;17:1749727. doi: 10.3389/fimmu.2026.1749727 (PMC12894041; doi:10.3389/fimmu.2026.1749727)
Supplement: Supplementary file 2 [file Table1.docx]

Supplementary Table 1. Basic information of patients with osteoarthritis

| **Basic information** | **OA**  **（n=30）** |
| --- | --- |
| Age (years) | 65.67± 12.68 |
| Gender（n/%） |  |
| Female | 24(80.00) |
| Male | 6(20.00) |
| Course of disease(year) | 10.00(7.00, 17.00) |
| BMI(kg/m^2^) | 22.65±2.81 |
| Combined diseases |  |
| Hypertension | 8(26.67%) |
| Diabetes | 6(20.00%) |
| Gastritis | 5(16.67%) |
| Combined medication |  |
| Angiotensin II receptor antagonist | 4(13.33%) |
| Calcium channel blocker | 3(10.00%) |
| Biguanides | 5(16.67%) |
| Sodium-glucose co-transporter 2 inhibitor | 2(6.67%) |
| Alpha-glucosidase inhibitor | 3(10.00%) |
| Proton pump inhibitor | 4(13.33%) |
