# Supplementary material for: Xinfeng capsule improves hyperinflammation-associated hypercoagulability and self-perception in osteoarthritis by regulating KLF4 through METTL14-mediated m6A modification of lncRNA MEG3
Source: Front Immunol. 2026 Jan 29;17:1749727. doi: 10.3389/fimmu.2026.1749727 (PMC12894041; doi:10.3389/fimmu.2026.1749727)
Supplement: Supplementary file 3 [file Table2.docx]

| Items(LHS➪RHS） | Support(%) | Confidence(%) | Lift |
| --- | --- | --- | --- |
| {XFC}➪{VAS↓} | 70.90 | 92.23 | 1.00 |
| {XFC}➪{hs-CRP↓} | 70.15 | 91.26 | 1.05 |
| {XFC}➪{ESR↓} | 69.40 | 90.29 | 1.01 |
| {XFC}➪{APTT↑} | 68.66 | 89.32 | 1.04 |
| {XFC}➪{RE↑} | 61.94 | 80.58 | 1.06 |
| {XFC}➪{PT↑} | 61.19 | 79.61 | 1.06 |
| {XFC}➪{SBS↓} | 61.19 | 79.61 | 1.03 |
| {XFC}➪{TT↓} | 61.19 | 79.61 | 1.04 |
| {XFC}➪{SAS↓} | 60.45 | 78.64 | 1.03 |
| {XFC}➪{SDS↓} | 58.21 | 75.73 | 1.00 |
| {XFC}➪{SDSSD↓} | 57.46 | 74.76 | 1.06 |
| {XFC}➪{GH↑} | 44.78 | 58.25 | 1.04 |
| {XFC}➪{RP↑} | 44.03 | 57.28 | 1.01 |
| {XFC}➪{PLT↓} | 41.79 | 54.37 | 1.03 |
| {XFC}➪{VT↑} | 39.55 | 51.46 | 0.97 |
| {XFC}➪{BP↑} | 36.57 | 47.57 | 1.01 |
| {XFC}➪{FBG↑} | 34.33 | 44.66 | 0.88 |

Supplementary Table 2 Association rule analysis under the condition of support of

30% and confidence of 40%.
